# Signal-induced NLRP3 phase separation initiates inflammasome activation

**DOI:** 10.1038/s41422-025-01096-6

**Published:** 2025-04-01

**Authors:** Gonglu Zou, Yuluan Tang, Jie Yang, Shuo Fu, Yuheng Li, Xuanyao Ren, Nanhai Zhou, Wenlong Zhao, Juyi Gao, Ziran Ruan, Zhengfan Jiang

**Affiliations:** 1 Key Laboratory of Cell Proliferation and Differentiation of the Ministry of Education, School of Life Sciences, Peking University, Beijing, China; 2 Peking-Tsinghua Center for Life Sciences, Peking University, Beijing, China

**Keywords:** NOD-like receptors, Stress signalling

## Abstract

NLRP3 inflammasome is activated by diverse stimuli including infections, intracellular and environmental irritants. How NLRP3 senses these unrelated stimuli and what activates NLRP3 remain unknown. Here we report that signal-dependent NLRP3 phase separation initiated its activation, in which the palmitoyltransferase ZDHHC7-mediated tonic NLRP3 palmitoylation and an IDR region in the FISNA domain of NLRP3 play important roles. Moreover, three conserved hydrophobic residues in the IDR critically mediate multivalent weak interactions. NLRP3-activating stimuli including K^+^ efflux and NLRP3-interacting molecules imiquimod, palmitate, and cardiolipin all cause NLRP3 conformational change and induce its phase separation and activation in cells and/or in vitro. Surprisingly, amphiphilic molecules like di-alcohols used to inhibit biomolecular phase separation and chemotherapeutic drugs doxorubicin and paclitaxel activate NLRP3 independently of ZDHHC7 by directly inducing NLRP3 phase separation. Mechanistically, amphiphilic molecules decrease the solubility of both palmitoylated and non-palmitoylated NLRP3 to directly induce its phase separation and activation while NLRP3 palmitoylation reduces its solubility to some extent without activation. Therefore, ZDHHC7-mediated NLRP3 palmitoylation in resting cells licenses its activation by lowering the threshold for NLRP3 phase separation in response to any of the diverse stimuli whereas NLRP3 solubility-reducing molecules like di-alcohols and chemotherapeutic drugs activate NLRP3 directly. The signal-induced NLRP3 phase separation likely provides the simplest and most direct mechanistic basis for NLRP3 activation.

## Introduction

The nucleotide-binding oligomerization domain-like receptor (NLR) family pyrin domain containing (NLRP)3 inflammasome is activated by diverse stimuli, the unifying factor of which is that they all induce cellular stress and is then sensed by NLRP3.^1–5^ Activated NLRP3 oligomerizes and interacts with the adaptor protein apoptosis-associated speck-like protein containing a CARD (ASC); ASC then assembles and recruits caspase-1, leading to caspase-1 activation and pyroptosis.^1^ In addition, the NIMA-related kinase 7 (NEK7) oligomerizes with NLRP3 into a complex for ASC assembly in mouse cells.^6,7^ Dysregulated NLRP3 activation is involved in many inflammatory, metabolic, and neurodegenerative diseases.^3,8,9^ Exactly how NLRP3 senses cellular stress and what directly activates NLRP3 is unknown. Although hypotheses propose an unidentified 2^nd^ messenger or yet-to-be-confirmed ones including mtDNA, reactive oxygen species (ROS), and K^+^ to activate NLRP3, none is suitable for all stimuli and lacks the mechanism of action.

Macromolecular phase separation regulates cellular processes by controlling molecule concentrations or sequestrating unwanted proteins.^10,11^ This biophysical response to intracellular changes is used for sensing and is faster than transcriptional or translational activation. One of the fundamental functions of phase separation is to respond to various intracellular stresses.^10,12^ It’s well established that electrostatic and hydrophobic interactions are important drivers for protein phase separation mediated by modular domains or intrinsically disordered region (IDR), which provides weak multivalent intermolecular interaction.^10,12,13^ Whether proteins phase separate or not is regulated by various factors influencing intermolecular interactions including temperature, salt concentration, protein concentration, and its post-translational modifications such as phosphorylation or ubiquitination. Protein palmitoylation is the only reversible lipidation that usually helps cytosolic proteins target distinct cytomembranes, catalyzed by palmitoyltransferases that contain a catalytic DHHC (asp-his-his-cys) tetrapeptide and is removed by acyl protein thioesterases.^14,15^ Recently, phase separation of ferroptosis suppressor protein-1 (FSP1) was found to promote ferroptosis dependent on the myristoylation of FSP1.^16^ Although previous work observed NLRP3 puncta formation upon activation,^17–19^ the role of phase separation in NLRP3 activation has been neglected and no protein palmitoylation-regulated phase separation has been reported.

In this study, we found that upon activation, NLRP3 phase separates in cells to form aggregates with liquid-like properties, which was dependent on ZDHHC7-mediated palmitoylation and an IDR in the FISNA domain of NLRP3 in which three conserved hydrophobic residues were crucial. We demonstrated that NLRP3 aggregation, a biophysical response, was induced by all tested NLRP3-activating stimuli including K^+^ efflux, imiquimod (IMQ), palmitate, and the mitochondrial lipid cardiolipin. Interestingly, we found that amphiphilic molecules including di-alcohols and chemotherapeutic drugs activated NLRP3 directly by inducing its phase separation in a ZDHHC7-independent way. We showed that there are at least three modes of action by signal-induced NLRP3 phase separation. The first one involves intracellular changes, such as ion efflux or influx, which induce NLRP3 conformational changes, thereby triggering phase separation. The second one is mediated by interactions with molecules like cardiolipin, palmitate, and IMQ, which also induce NLRP3 conformational changes leading to phase separation. Both of these modes are highly dependent on ZDHHC7-mediated NLRP3 palmitoylation. The third mode involves amphiphilic molecules, including amphiphilic diols and chemotherapy drugs, which induce NLRP3 phase separation by reducing its solubility, independent of ZDHHC7.

## Results

### ZDHHC7 is required for NLRP3 activation

**Fig. 1:**
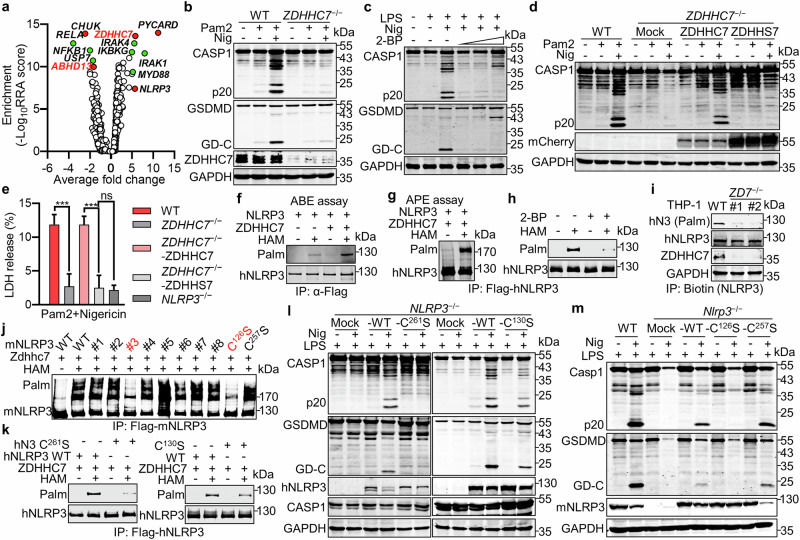
ZDHHC7-mediated NLRP3 palmitoylation is required for NLRP3 activation. **a** Enriched genes by the genome-wide CRISPR-Cas9 screening. Genes in NF-κB pathway are shown as green spots, genes known in NLRP3 inflammasome are shown as red spots, and genes (red font) with no known function in NLRP3 pathway are shown as red spots. **b** NLRP3 activation in WT or *ZDHHC7*^–/–^ THP-1 cells. Cells were pretreated with 10 ng/mL Pam2CSK4 for 3 h, followed by 4 μM nigericin treatment for another 1 h. **c** NLRP3 activation in THP-1 cells pretreated with 2-BP or not before immunoblotting. Cells were pretreated with 1 μg/mL LPS for 2 h and then with the 2-BP (2.5, 5, 10 μM) for 1 h, followed by 4 μM nigericin treatment for another 1 h. **d** NLRP3 activation in the WT, *ZDHHC7*^–/–^ or *ZDHHC7*^–/–^ THP-1 cells reconstituted with ZDHHC7- or ZDHHS7-mCherry. ZDHHS7, S-acyltransferase catalytic mutant C^160^S. Cells were treated as in (**b**). **e** LDH analysis in (**d**). **f**, **g** Palmitoylation of Flag-hNLRP3 in HEK293T cells with ZDHHC7 expression was detected by ABE (**f**) or APE assay (**g**). **h** HeLa cells stably expressing Flag-hNLRP3 were treated overnight with 2-BP (100 μM) as indicated. The hNLRP3 palmitoylation level was detected by ABE assay. **i** Palmitoylation of endogenous NLRP3 in WT or *ZDHHC7*^–/–^ THP-1 cells detected by ABE assay. **j** B16 cells expressing Flag-mNLRP3 with indicated combined mutations (Supplementary information, Fig. S3e). The mNLRP3 palmitoylation level was detected by APE assay. **k** Palmitoylation of Flag-hNLRP3-WT, Flag-hNLRP3-C^130^S, or Flag-hNLRP3-C^261^S in HEK293T cells with ZDHHC7 expression was detected by ABE assay. **l** NLRP3 activation in *NLRP3*^–/–^ or *NLRP3*^–/–^ THP-1 cells reconstituted with indicated NLRP3. Cells were pretreated with 1 μg/mL LPS for 3 h, followed by 4 μM nigericin treatment for another 1 h. **m** mNLRP3 activation in WT or *Nlrp3*^–/–^ immortalized BMDM (iBMDM) cells reconstituted with indicated Flag-mNLRP3. Cells were pretreated with 1 μg/mL LPS for 3 h, followed by 6 μM nigericin treatment for another 1 h. Statistical significance was indicated as follows: ns, not significant, ***P < 0.001.

We constructed a THP-1 cell line with a heterozygous gain-of-function mutation in hNLRP3 (R^262^W) using the CRISPR-Cas9 system. The expression level of NLRP3 was comparable between THP-1-R^262^W and THP-1 cells (Supplementary information, Fig. S1a, b). THP-1-R^262^W cells can be activated by TLR4 agonist lipopolysaccharide (LPS) or TLR2/6 agonist Pam2CSK4 alone without the second signal^20,21^ (Supplementary information, Fig. S1c, d). This mutation in patients causes autoinflammatory diseases,^22,23^ equivalent to R^258^W autoactive mutant of mNLRP3.^20^ We performed genome-wide CRISPR-Cas9 screening using this 2^nd^ signal-independent THP-1-R^262^W cells to search for genes important for NLRP3 priming as previously reported, and identified genes involved in the nuclear factor (NF)-κB pathway as expected. Among enriched genes, *ZDHHC7*^24^ and *ABHD13*,^25^ encoding a palmitoyltransferase and a putative depalmitoyltransferase, respectively, were most intriguing (Fig. 1a). ZDHHC7 deficiency in THP-1-R^262^W cells indeed suppressed LPS-induced caspase-1 activation, lactate dehydrogenase (LDH) release (Supplementary information, Fig. S1e, f), and pyroptotic cell death (Supplementary information, Fig. S1g). *ZDHHC7*^−/−^ THP-1 cells showed even severer effects to NLRP3-activating stimuli (Fig. 1b; Supplementary information, Fig. S1h–l), without affecting the induction of genes including pro-interleukin (IL)-1β by various TLR ligands (Supplementary information, Fig. S1m, n). Moreover, ZDHHC7 is required for priming-independent activation of NLRP3 in THP-1 cells stimulated by nigericin or IMQ alone (Supplementary information, Fig. S1o). Thus, ZDHHC7 is required for both priming-dependent and -independent NLRP3 activation, suggesting that it functions before the priming step of inflammasome activation.

### ZDHHC7 mediates a continuous NLRP3 palmitoylation

Treatment of S-acyltransferase inhibitors 2-bromopalmitate (2-BP) or cerulenin, but not the farnesyl transferase inhibitor FTI-276 or geranyl transferase inhibitor GGTI-2133, blocked NLRP3 activation (Fig. 1c; Supplementary information, Fig. S2a, b), indicating that the S-acyltransferase activity of ZDHHC7 is important. Consistently, *ZDHHC7*^−/−^ THP-1 cells reconstituted with wild-type (WT) ZDHHC7 but not ZDHHS7 (S-acyltransferase catalytic mutant C^160^S) fully restored NLRP3 activation (Fig. 1d, e). The acyl-biotin exchange (ABE) assay and acyl-polyethylene glycol exchange (APE) assay for protein palmitoylation detection revealed that NLRP3 palmitoylation in cells occurred without any treatment, while ectopically expressed ZDHHC7, but not ZDHHS7, substantially promoted NLRP3 palmitoylation (Fig. 1f, g; Supplementary information, Fig. S2c). Moreover, the palmitic acid analog 17-octadecynoic acid (17-ODYA) conjugated to biotin-PEG3-azide was used as a metabolic label (hereinafter as click chemistry); this assay confirmed the palmitoylation of NLRP3 (Supplementary information, Fig. S2d). Both ABE assay and click chemistry showed that 2-BP or cerulenin, but not FTI-276 or GGTI-2133, impaired NLRP3 palmitoylation (Fig. 1h; Supplementary information, Fig. S2e–g), and abolished NLRP3 activation in THP-1 cells (Fig. 1c; Supplementary information, Fig. S2a, b). Consistently, the constitutive NLRP3 palmitoylation was significantly reduced in *ZDHHC7*^−/−^ cells compared with the WT cells (Fig. 1i; Supplementary information, Fig. S2h–j). Thus, ZDHHC7 mediates a continuous NLRP3 palmitoylation in cells functioning before the priming step of activation.

A recent study showed that ZDHHC12 palmitoylates and promotes NLRP3 degradation in lysosomes to negatively regulate NLRP3.^26^ We considered that NLRP3 palmitoylation by ZDHHC7 or ZDHHC12 occurs in different stages and exerts distinct effects, as ZDHHC7 did not affect NLRP3 protein stability (Supplementary information, Fig. S2k, l). Moreover, NLRP3 palmitoylation increased after nigericin treatment in THP-1 cells (Supplementary information, Fig. S2m), but kept unchanged in *PYCARD*^−/−^ THP-1, or ASC-null HeLa cells (Supplementary information, Fig. S2j, n), indicating that whereas ZDHHC12-mediated NLRP3 palmitoylation is a negative feedback regulation, ZDHHC7-mediated persistent NLRP3 palmitoylation is a prerequisite for NLRP3 activation in response to nigericin.

ZDHHC7 was localized to the *trans*-Golgi network (TGN) and lysosomes (Supplementary information, Fig. S2o). Because ZDHHC7 palmitoylated NLRP3 without any stimulation, we speculated that ZDHHC7 interacts with NLRP3 in a “hit-and-run” manner. This speculation was supported by the lack of evident ZDHHC7–NLRP3 colocalization before and after stimulation (Supplementary information, Fig. S2p). Nevertheless, the enhanced interaction between overexpressed NLRP3 and ZDHHC7, but not ZDHHS7, suggested that catalytic activity strengthens or prolongs their interaction (Supplementary information, Fig. S2q).

### ZDHHC7 palmitoylates hNLRP3 at Cys^130^ and Cys^261^ and mNLRP3 at Cys^126^

NLRP3 palmitoylation was sensitive to hydroxylamine, indicating the modification of cysteine residues^27^ (Fig. 1f, g). To map the palmitoylation sites of hNLRP3, nine combined hNLRP3 mutations substituting a total of 45 cysteines with serines were made and analyzed (Supplementary information, Fig. S3a). hNLRP3 palmitoylation was substantially reduced on combined mutants #1, #2, and #6 (Supplementary information, Fig. S3b). Individual mutation revealed that C^130^ and C^261^ to be hNLRP3 palmitoylation sites (Supplementary information, Fig. S3c, d) and C^126^ alone (corresponding to the C^130^ of hNLRP3) to be the mNLRP3 palmitoylation site (Fig. 1j; Supplementary information, Fig. S3e). ABE assay revealed C^261^ is the main site of hNLRP3 palmitoylation (Fig. 1k). Consistently, hNLRP3-C^261^S exhibited significantly reduced palmitoylation (Supplementary information, Fig. S3f) and impaired inflammasome activation (Fig. 1l). mNLRP3-C^126^S failed to activate while mNLRP3-C^257^S (corresponding to the C^261^ of hNLRP3) showed normal activation (Fig. 1m). C^130^ in hNLRP3 is conserved in all identified NLRP3 orthologues (Supplementary information, Fig. S3g) and C^130^ and C^261^ are localized on the surface of inactive hNLRP3 (Supplementary information, Fig. S3h, i), thus structurally allowing for ZDHHC7–NLRP3 interaction in resting cells.

### ABHD13 depalmitoylates and negatively regulates NLRP3

We next examined the role of ABHD13 in NLRP3 activation. Protein sequence alignment predicts S^193^, D^268^, and H^298^ to be the catalytic sites for its enzymatic activity.^28^ *ABHD13*^−/−^ THP-1 cells exhibited enhanced NLRP3 activation, IL-1β secretion, and LDH release (Supplementary information, Fig. S4a–d). Similar results were obtained in *Abhd13*^−/−^ bone marrow-derived macrophages (BMDMs) (Supplementary information, Fig. S4e–h). *ABHD13*^−/−^ THP-1 cells reconstituted with ABHD13, but not the catalytic mutants S^193^A/D^268^A/H^298^A (SDH) restored normal NLRP3 activation, IL-1β secretion, and LDH release (Supplementary information, Fig. S4i, j). Consistently, ABE assay and click chemistry showed that the continuous NLRP3 palmitoylation was significantly enhanced in *ABHD13*^−/−^ cells (Supplementary information, Fig. S4k–m). ABHD13-mediated depalmitoylation occurred at C^130^ and C^261^, but not C^837^ or C^838^, which has been reported to be depalmitoylated by ABHD17A^29^ (Supplementary information, Fig. S4n). These results indicated that ABHD13 depalmitoylates NLRP3 and negatively regulates its activation.

### ZDHHC7 is important for NLRP3 activation in vivo

**Fig. 2:**
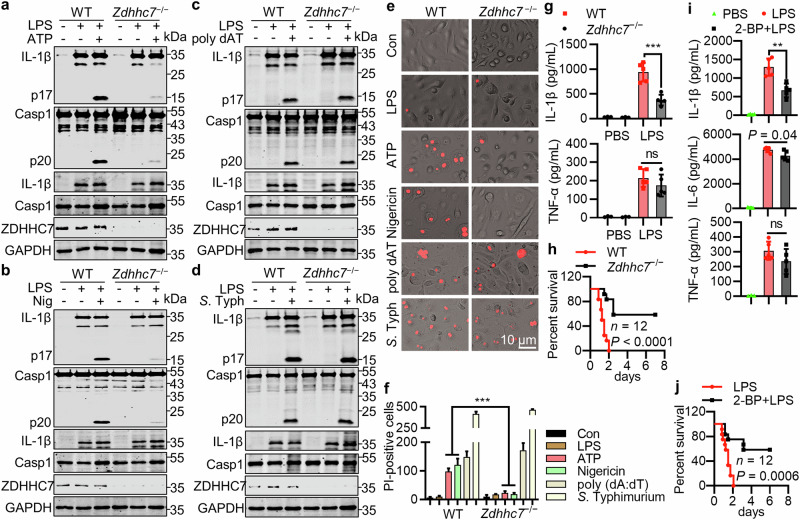
ZDHHC7 is required for NLRP3 inflammasome activation in vivo. **a**, **b** mNLRP3 activation in WT or *Zdhhc7*^−/−^ BMDMs by ATP (**a**) or nigericin (**b**) treatment. Cells were pretreated with 1 μg/mL LPS for 3 h, followed by 5 mM ATP or 6 μM nigericin treatment for another 1 h. **c**, **d** Aim2 (**c**) or Nlrc4 (**d**) activation in WT and *Zdhhc7*^−/−^ BMDMs by poly(dA:dT) transfection or *S*. Typhimurium infection. Cells were pretreated with 1 μg/mL LPS for 3 h, followed by 1 μg/mL poly(dA:dT) transfection for 4 h or *S*. Typhimurium (MOI = 0.5) infection for 1 h. **e**, **f** PI staining (**e**) and percentage of PI-positive cells (**f**) in WT and *Zdhhc7*^−/−^ BMDMs treated as in (**a**–**d**). **g** WT or *Zdhhc7*^–/–^ mice (*n* = 5) were intraperitoneally injected with PBS or LPS (22.5 mg/kg) for 12 h. IL-1β and TNF-α in sera were detected by enzyme-linked immunosorbent assay (ELISA) analysis. **h** WT or *Zdhhc7*^–/–^ mice (*n* = 12) were intraperitoneally injected with LPS (22.5 mg/kg); survival was calculated using the Mantel–Cox test. **i** WT mice (*n* = 5) were intraperitoneally injected with vehicle or 2-bromopalmitate (2-BP, 50 mg/kg) twice 24 h or 1 h before experiments, then subjected to intraperitoneal injection of PBS or LPS (22.5 mg/kg) for 12 h. IL-1β, TNF-α, and IL-6 in sera were detected by ELISA analysis. **j** WT mice (*n* = 12) were intraperitoneally injected with 2-BP and LPS as in (**i**), and survival was calculated using the Mantel–Cox test. Statistical significance was indicated as follows: ns, not significant, **P < 0.01, ***P < 0.001.

The importance of mouse NLRP3 palmitoylation for its activation was confirmed in peritoneal macrophages, pulmonary fibroblasts (Supplementary information, Fig. S5a–d), and BMDMs (Fig. 2a, b; Supplementary information, Fig. S5e) from *Zdhhc7*^−/−^ mice. *Zdhhc7*^−/−^ BMDMs showed normal activation of Aim2 or Nlrc4 inflammasome by poly (dA:dT) treatment or *Salmonella* Typhimurium infection, respectively (Fig. 2c, d). These results were consistent with the observations of pyroptotic cell death (Fig. 2e, f). To investigate the physiological function of ZDHHC7, we established an LPS-induced sepsis model that involves mNLRP3 activation.^24^ After LPS challenge, serum IL-1β but not tumor necrosis factor (TNF)-α level was significantly lower in *Zdhhc7*^−/−^ mice than in WT mice (Fig. 2g). Consistently, *Zdhhc7*^−/−^ mice showed significantly improved survival (Fig. 2h). Thus, ZDHHC7 is required for mNLRP3 activation in vivo. Moreover, 2-BP-pretreated mice produced less IL-1β, but not TNF-α or IL-6 in sera (Fig. 2i). Consistent with these findings, 2-BP significantly improved the survival of LPS-challenged mice (Fig. 2j). These data indicated that pharmacological inhibition of NLRP3 palmitoylation may be used for the intervention of NLRP3-implicated diseases.

To test this hypothesis, we first constructed chimeric proteins in which GFP was fused to an hNLRP3 (residues 254–268) fragment (“GFP-C^261^”) or to the same fragment but with C^261^S mutation (“GFP-S^261^”) to determine whether C^261^-containing peptides can inhibit NLRP3 activation (Supplementary information, Fig. S5f). GFP-C^261^, but not GFP-S^261^, interacted with ZDHHC7 (Supplementary information, Fig. S5g). Furthermore, when co-overexpressed with ZDHHC7, GFP-C^261^ exhibited much stronger palmitoylation compared with GFP-S^261^ (Supplementary information, Fig. S5h). Next, we constructed THP-1 cells stably expressing GFP, GFP-C^261^, or GFP-S^261^ and found that GFP-C^261^-expressing cells showed substantially reduced NLRP3 activation, compared with GFP- or GFP-S^261^-expressing cells upon treatment (Supplementary information, Fig. S5i). Finally, synthetic C^261^-containing peptides were assessed to determine their effect on NLRP3 activation. Whereas a 7-AA peptide showed no effect, 11-AA and 15-AA peptides strongly inhibited NLRP3 activation in a dose-dependent manner, consistent with the increased palmitoylation levels and enhanced interaction with ZDHHC7 (Supplementary information, Fig. S5j–n). Thus, C^261^-containing peptides may have therapeutic value as competitive inhibitors of NLRP3.

### NLRP3 forms aggregates after nigericin treatment

**Fig. 3:**
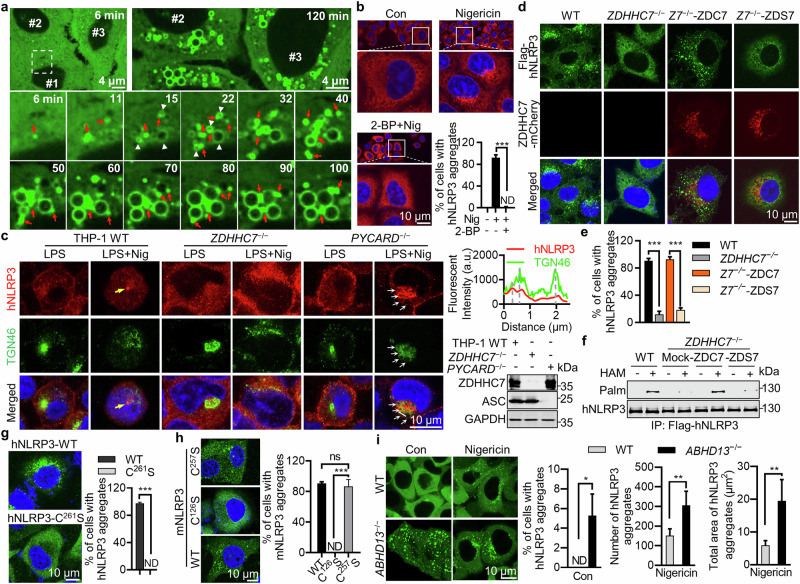
ZDHHC7-mediated NLRP3 palmitoylation is required for NLRP3 aggregation. **a** Images of HeLa cells stably expressing mNG-NLRP3 were taken at the indicated times after 8 μM nigericin treatment. Red arrows represent NLRP3 aggregates and white triangles represent vesicles. Scale bars, 4 μm. **b** Images or statistical histogram of HeLa cells stably expressing Flag-hNLRP3 pretreated with 50 μM 2-BP overnight followed by 8 μM nigericin treatment for another 1 h, and immunostained with anti-Flag antibody. Scale bar, 10 μm. The percentage of cells with NLRP3 condensates was quantified from at least 100 cells (*n* = 3, mean ± s.d., two-sided *t*-test). ND not detectable. **c** Images (left) and colocalization analysis (right) of WT, *ZDHHC7*^−/−^, and *PYCARD*^−/−^ THP-1 cells pretreated with 1 μg/mL LPS for 3 h alone, or followed by 4 μM nigericin treatment for another 1 h, immunostained with anti-hNLRP3 or anti-TGN46 antibody. Scale bar, 10 μm. Quantitative analysis of co-localization along a white line was shown. White arrows, colocalized TGN46 and hNLRP3. Yellow arrows, NLRP3-NEK7-ASC specks. ZDHHC7 and ASC expression was analyzed by western blotting assay using antibodies against ZDHHC7, ASC and GAPDH (right). **d** HeLa WT, *ZDHHC7*^–/–^, and *ZDHHC7*^–/–^ cells reconstituted with ZDHHC7-mCherry (ZDC7) or ZDHHS7-mCherry (ZDS7) were stimulated with 8 μM nigericin for 1 h, and immunostained with anti-Flag antibody before imaging. **e** Percentage of cells with hNLRP3 aggregates in (**d**). **f** Palmitoylation of Flag-hNLRP3 in WT, *ZDHHC7*^–/–^, or *ZDHHC7*^–/–^ HeLa cells reconstituted with ZDHHC7- or ZDHHS7-mCherry was detected by ABE assay. **g** Images (left) or percentage of cells with hNLRP3 aggregates (right) in HeLa cells stably expressing indicated Flag-hNLRP3 after nigericin treatment. Cells were treated with 8 μM nigericin for 1 h, immunostained with anti-Flag antibody. **h** Images (right) or percentage of cells with mNLRP3 aggregates (left) in HeLa cells stably expressing indicated Flag-mNLRP3 mutants after nigericin treatment. Cells were treated with 8 μM nigericin for 1 h, and immunostained with anti-Flag antibody. **i** Images (left) and statistical diagram (right) of mNG-hNLRP3 aggregates in WT and *ABHD13*^−/−^ HeLa cells left untreated (Con) or treated with 8 μM nigericin for 1 h before live cell imaging. Statistical significance was indicated as follows: ns, not significant, *P < 0.05, **P < 0.01, ***P < 0.001.

Upon stimulation, NLRP3 is recruited to dispersed trans-Golgi network (dTGN)^18,19,30^ or endosome-derived vesicles,^31^ where it becomes activated. Since protein palmitoylation usually helps cytosolic proteins target cytomembranes,^14,15^ we next tested if NLRP3 palmitoylation is important for its vesicle targeting. We found that stably expressed N-terminal mNeonGreen-, Flag-, or HA-tagged or C-terminal GFP-tagged hNLRP3 was evenly distributed in HeLa cells, but obviously aggregated upon treatment (Supplementary information, Fig. S6a), which was confirmed by using an anti-hNLRP3 antibody to detect untagged or endogenous hNLRP3 (Supplementary information, Fig. S6b). After nigericin treatment, a portion of NLRP3 was recruited to dTGN and endosome-derived vesicles (Supplementary information, Fig. S6c). Using time-lapse microscopy and HeLa cells stably expressing mNG-hNLRP3 (mNeonGreen-hNLRP3), we found that upon nigericin treatment, NLRP3 aggregation (red arrows) and vesicle-recruitment (white triangles) simultaneously occurred. NLRP3 aggregates always attached to vesicles, seemingly distributing NLRP3 over the surface of these vesicles to be visible (Fig. 3a; Supplementary information, Fig. S6d and Video S1). Interestingly, NLRP3 vesicles became larger with time and vigorously communicated with each other by NLRP3 aggregates (Supplementary information, Fig. S6d, white arrows and yellow triangles). These NLRP3 aggregates (greater than 500 nm in diameters) clearly demonstrated liquid-like properties with dynamic fissions and fusions, indicative of liquid-liquid phase separation (LLPS) of NLRP3 upon activation. 2-BP (Fig. 3b) or cerulenin but not FTI-276 or GGTI-2133 (Supplementary information, Fig. S6e) abolished nigericin-induced NLRP3 aggregation and vesicle recruitment. Two anti-NLRP3 antibodies were used to further investigate the endogenous localization of NLRP3 (Supplementary information, Fig. S6f, g). Consistently, NLRP3 in *ZDHHC7*^−/−^ THP-1 cells did not aggregate after nigericin treatment, in contrast to that in the WT cells (Fig. 3c). Expression of ZDHHC7, but not ZDHHS7, in *ZDHHC7*^−/−^ HeLa cells restored hNLRP3 palmitoylation, aggregation and vesicle recruitment (Fig. 3d–f). Similar results were observed for mNLRP3 in *Zdhhc7*^−/−^ iBMDM cells (Supplementary information, Fig. S6h, i). Neither hNLRP3-C^261^S nor mNLRP3-C^126^S mutant aggregated or formed vesicles probably due to altered conformation (C^261^S) similar to R^262^ mutations or required polybasic region and the adjacent palmitoylation for protein membrane attachment as the “two-signal hypothesis” suggests,^32^ whereas mNLRP3-C^257^S (corresponding to human C^261^S) aggregated normally (Fig. 3g, h; Supplementary information, Fig. S6j). Moreover, NLRP3 stably expressed in *ABHD13*^−/−^ but not in the WT cells spontaneously aggregated; stimulation led to enhanced aggregation (Fig. 3i). These results collectively indicated that ZDHHC7-mediated NLRP3 palmitoylation is required for NLRP3 aggregation and activation.

### Polybasic regions and vesicle localization were dispensable for NLRP3 activation

**Fig. 4:**
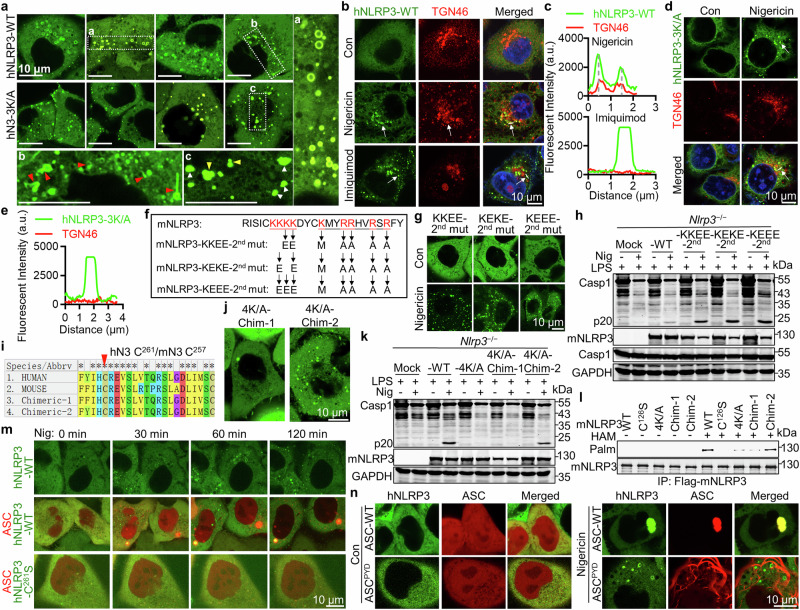
Condensation but not vesicle localization is required for NLRP3 activation. **a** Live cell images of HeLa cells stably expressing mNeonGreen (mNG)-hNLRP3 or mNeonGreen-hNLRP3-3K/A mutant after 1 h treatment with 8 μM nigericin. Scale bars, 10 μm. **b**, **c** Images (**b**) and colocalization analysis (**c**) of HeLa cells stably expressing mNG-hNLRP3 after 8 μM nigericin or 40 μg/mL imiquimod for 1 h, immunostained with anti-TGN46 antibody. Quantitative analysis of co-localization along a white line was shown. **d**, **e** The same as (**b**,** c**) except HeLa cells stably expressing mNG-hNLRP3-3K/A were used. **f** Sequence information of mNLRP3-KKEE-2^nd^, mNLRP3-KEKE-2^nd^ and mNLRP3-KEEE-2^nd^ mutants. **g** Live cell images of HeLa cells stably expressing indicated mNLRP3 mutants left untreated or treated with 8 μM nigericin for 1 h. **h** mNLRP3 activation in *Nlrp3*^–/–^ iBMDM cells reconstituted with indicated mNLRP3 mutants. Cells were pretreated with 1 μg/mL LPS for 3 h, followed by 6 μM nigericin for another 1 h. **i** Sequence information of hNLRP3, mNLRP3, Chimeric mNLRP3-4K/A-1 and Chimeric mNLRP3-4K/A-2. **j** Live cell images of HeLa cells stably expressing indicated Chimeric mNLRP3-4K/A in **i**. Cells were treated with 8 μM nigericin for 1 h. **k** mNLRP3 activation in *Nlrp3*^–/–^ iBMDM cells reconstituted with the indicated mNLRP3 mutants. Cells were pretreated with 1 μg/mL LPS for 3 h, followed by 6 μM nigericin for another 1 h. **l** Palmitoylation of the WT, C^126^S, 4 K/A, Chim-1 or Chim-2 mNLRP3 in B16 cells detected by the ABE assay. **m** Images of HeLa cells stably expressing mNG-hNLRP3, mNG-hNLRP3 and dox-induced mScarlet-ASC, or mNG-hNLRP3-C^261^S and dox-induced mScarlet-ASC at the indicated times after 8 μM nigericin treatment. Cells were treated with 1 μg/mL dox for 6 h before imaging. Scale bar, 10 μm. **n** Images of HeLa cells stably expressing mNG-hNLRP3 and mScarlet-hASC or mScarlet-hASC^PYD^ (1–91 AA). Cells were left untreated (Con, left) or treated with 8 μM nigericin (right) for 1 h before live cell imaging. Scale bar, 10 μm.

Previous work reported that a polybasic K^127^KKK^130^ (4 K) region in mNLRP3 is important for its membrane recruitment and activation.^18^ As protein palmitoylation helps proteins attach to membranes and a “two-signal hypothesis” indicates both covalently linked fatty acids and a polybasic region are required for protein’s stable membrane recruitemt,^32^ we hypothesized that NLRP3 requires both palmitoylation and its polybasic region for vesicle recruitment. To test this, mNG-hNLRP3-3K/A mutant in which all three lysines in the K^131^MKK^134^ corresponding to the mNLRP3-K^127^KKK^130^ (4 K) region replaced by alanines (A^131^AAA^134^, hereinafter hNLRP3-3K/A) was constructed and transiently expressed in HEK293T cells, which showed slightly decreased NLRP3 palmitoylation (Supplementary information, Fig. S7a). In contrast to the WT hNLRP3, hNLRP3-3K/A did not show any vesicle recruitment (Fig. 4a, yellow and white triangles), consistent with previous works.^17,18^ However, hNLRP3-3K/A aggregated and was activated normally after nigericin treatment after being reconstituted in *NLRP3*^*−/−*^ THP-1 cells (Fig. 4a; Supplementary information, Fig. S7b, c). This observation agreed with a previous work.^17^ Interestingly, IMQ only induced NLRP3 aggregation but not vesicle-recruitment, similar to hNLRP3-3K/A aggregation induced by nigericin (Fig. 4b–e). These results strongly indicated that NLRP3 vesicle recruitment is not required for its downstream activation. In nigericin-treated HeLa cells stably expressing mNG-tagged hNLRP3 or hNLRP3-3K/A, co-staining NLRP3 with TGN46 or the lipid-interacting BODIPY dye revealed that TGN46-colocalized NLRP3 vesicles were essentially all co-stained with BODIPY. However, aggregates formed by hNLRP3 or hNLRP3-3K/A did not colocalize with TGN46 and were completely separated from BODIPY-stained vesicles (Fig. 4b–e; Supplementary information, Fig. S7d), indicating that NLRP3 aggregates are free of cytomembrane. As previous work reported,^18^ mNLRP3-4K/A mutant was not activated in the reconstituted *Nlrp3*^−/−^ iBMDM cells (Supplementary information, Fig. S7e), neither did it aggregate nor attach to vesicles in HeLa cells stably expressing mNLRP3-4K/A whereas mNLRP3, hNLRP3 or hNLRP3-3K/A all aggregated in HeLa cells after nigericin treatment (Supplementary information, Fig. S7f). Mutating the second polybasic region in which all the basic residues were replaced by methionine or alanines (hereinafter mNLRP3-2^nd^ mut) kept normal mNLRP3 activation (Supplementary information, Fig. S7g). To further determine the role of the polybasic region (K^127^KKK^130^) of mNLRP3 in its activation, mNLRP3-KKEE-2^nd^, mNLRP3-KEKE-2^nd^, and mNLRP3-KEEE-2^nd^ mutants, in which indicated lysines were mutated to neutralize or even reverse the electric charge in this region in the mNLRP3-2^nd^ mutant (Fig. 4f), were constructed and stably expressed in HeLa cells. Surprisingly, all these mutants aggregated normally after nigericin treatment without vesicle recruitment as expected (Fig. 4g), and fully restored inflammasome activation in *Nlrp3*^−/−^ iBMDM (Fig. 4h). Again, aggregates formed by mNLRP3 or mNLRP3-KEEE-2^nd^ mutant did not colocalize with TGN46 and were entirely free of BODIPY staining (Supplementary information, Fig. S7h, i). These results confirmed that neither the polybasic regions nor vesicle recruitment is important for hNLRP3 and mNLRP3 activation. Since ZDHHC7 only palmitoylates mNLRP3 at C^126^ that is right before the 4 K region and NLRP3 palmitoylation is required for its aggregation and activation, we reasoned that this 4 K/A mutation, particularly the K^127^A mutation, may affect C^126^ palmitoylation. Indeed, palmitoylation was almost abolished in mNLRP3-4K/A mutant (Supplementary information, Fig. S7j–l), in contrast to partially reduced NLRP3 palmitoylation in hNLRP3-3K/A, as hNLRP3-C^261^ was the main palmitoylation site. Importantly, mNLRP3-K^127^A mutant significantly reduced its palmitoylation and activation to a similar extent as the mNLRP3-4K/A mutant (Supplementary information, Fig. S7l, m), but with normal recruitment to TGN46-enriched vesicles after nigericin treatment. This mutant did not form any condensates after IMQ treatment (Supplementary information, Fig. S7n, o). Similar to IMQ, ATP induced NLRP3 phase separation and activation without NLRP3 vesicle recruitment (Supplementary information, Fig. S7p, q). Moreover, an introduced palmitoylation site in a chimeric mNLRP3-4K/A with indicated residues nearby mNLRP3-C^257^ replaced by the corresponding residues in hNLRP3 (mNLRP3-4K/A-Chim2) rendered mNLRP3-4K/A sensitivity to nigericin again and revived this dead mutant (Fig. 4i–k). Consistently, mNLRP3-4K/A-Chim2 restored palmitoylation of mNLRP3-4K/A similar to that of WT mNLRP3 (Fig. 4l). Together, these results confirmed that polybasic regions and vesicle localization were dispensable for NLRP3 aggregation and activation.

Since NLRP3 vesicles were vividly observed in nigericin-treated ASC-deficient cells like HeLa or *PYCARD*^−/−^ iBMDM with ectopically expressed NLRP3,^18,19,30,31^ we tested what happens to NLRP3 vesicles in ASC-expressing cells. We found that although nigericin treatment dramatically induced NLRP3 vesicles, these NLRP3-posistive vesicles disappeared upon ASC expression in HeLa cells (Fig. 4m; Supplementary information, Video S2), indicating that NLRP3 vesicle recruitment is correlated with ASC deficiency in cells. However, when pyrin domain of ASC (ASC^PYD^) was expressed in cells, which forms filamentous structures without oligomerization,^18,33^ it only formed prominent filaments without affecting NLRP3 vesicles (Fig. 4n), indicating that the oligomerization ability of ASC is required for NLRP3 sequestration outside vesicles.

### NLRP3 phase separates upon activation

**Fig. 5:**
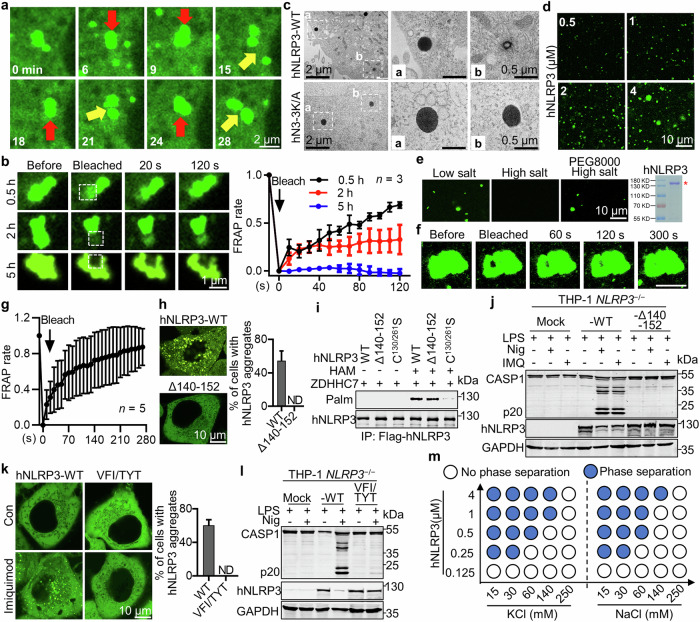
NLRP3 phase separates upon activation. **a** Images of HeLa cells stably expressing mNG-hNLRP3-3K/A were taken at the indicated times after 8 μM nigericin treatment. Red and yellow arrows represent the fusion and fission of NLRP3 aggregates, respectively. Scale bar, 2 μm. **b** FRAP of HeLa cells stably expressing mNG-hNLRP3, treated with nigericin for the indicated time. The white boxes indicate bleached sites. Scale bar, 1 μm. The normalized FRAP rate was shown on the right. s, second; *n* = 3. **c** HeLa cells stably expressing APEX-hNLRP3 -EGFP or APEX-hNLRP3-3K/A-EGFP (hN3-3K/A) were stimulated with 8 μM nigericin for 1 h before imaging by the transmission electron microscopy. The white boxes (**a**,** b**) indicate magnified regions of right images. Scale bars, as indicated. **d** In vitro NLRP3 droplet formation after diluting mNG-hNLRP3 protein to the indicated concentrations into a phase-separation buffer at room temperature (RT). **e** NLRP3 (1 μM) LLPS in low salt (60 mM KCl), high salt (250 mM KCl) or high salt with 1.75% PEG8000. Scale bar, 10 μm. Coomassie blue staining of purified mNG-hNLRP3 protein was shown on the right. **f** NLRP3 droplets formed as in **e**. Images of hNLRP3 aggregates before and after photobleaching. Scale bar, 10 μm. **g** Quantification of FRAP data in **f**. s, second; *n* = 5. **h** Live cell images (left) or percentage of cells with NLRP3 aggregates (right) in HeLa cells stably expressing mNG-hNLRP3 with indicated mutants after 40 μg/mL imiquimod treatment for 1 h. Scale bar, 10 μm. **i** Palmitoylation of the WT, Δ140–152, or C^130/261^S hNLRP3 in HEK293T cells with ZDHHC7 expression detected by the ABE assay. **j** NLRP3 activation in *NLRP3*^–/–^ THP-1 cells reconstituted with the indicated hNLRP3 mutants. Cells were pretreated with 1 μg/mL LPS for 3 h, followed by 4 μM nigericin or 30 μg/mL imiquimod for another 1 h. **k** Live cell images (left) or percentage of cells with NLRP3 aggregates (right) in HeLa cells stably expressing mNG-hNLRP3 or mNG-hNLRP3-VFI/TYT. Cells were treated with 40 μg/mL imiquimod for 1 h. **l** NLRP3 activation in *NLRP3*^–/–^ THP-1 cells reconstituted with hNLRP3 or VFI/TYT. Cells were pretreated with 1 μg/mL LPS for 3 h, followed by 4 μM nigericin for another 1 h. **m** Phase-separation diagram of hNLRP3, KCl and NaCl at the indicated concentrations at RT after 1 min in phase-separation buffer.

Proteins self-organize into droplets by LLPS of proteins,^10,11,34^ which allows them to be concentrated and contributes to the spatial and temporal regulation of their biochemical reactions. Agreeing with previous studies,^17–19^ activated NLRP3 rigorously formed puncta or aggregates with liquid-like properties, which was dependent on NLRP3 palmitoylation; we therefore hypothesized that palmitoylation promotes NLRP3 phase separation since it provides hydrophobicity to NLRP3 and it is well known that electrostatic and hydrophobic interactions are important for macromolecular phase separation.^10,12^ Using time-lapse microscopy and HeLa cells stably expressing mNG-hNLRP3-3K/A, we found that nigericin induced a robust NLRP3 condensation, in which condensates interacted with each other actively by fusions (red arrows) and fissions (yellow arrows) (Fig. 5a; Supplementary information, Video S3). Fluorescence recovery after photobleaching (FRAP) analysis showed that only early-state condensates exhibited FRAP (Fig. 5b; Supplementary information, Video S4), in contrast to late-state condensates (Fig. 5b; Supplementary information, Video S5), suggesting that the aggregated NLRP3 underwent a liquid-to-solid transition in cells 5 h after nigericin treatment as phase separated biomolecules usually do.^16,35–37^ We also utilized the ascorbate peroxidase 2 (APEX2) to trace NLRP3 condensation and found that stably expressed APEX-hNLRP3-GFP or APEX-hNLRP3-3K/A-GFP (APEX2 attached to hNLRP3 N-terminus) was evenly distributed in cytoplasm of HeLa cells, but phase separated rigorously after nigericin treatment. NLRP3 activation was not affected by the combined APEX and GFP tags (Supplementary information, Fig. S8a). Again, hNLRP3-3K/A only aggregated without vesicle recruitment, in sharp contrast to hNLRP3 (Supplementary information, Fig. S8b). Correlative Light and Electron Microscopy (CLEM) revealed NLRP3 condensates displaying strong concrete APEX signal with diameters typically around several hundred nanometers (Fig. 5c; Supplementary information, Fig. S8c) and aggregates formed by hNLRP3-3K/A were generally bigger than those formed by hNLRP3-WT, probably due to shared NLRP3 between aggregates and vesicles. NLRP3 vesicles with APEX signal outside of membrane were revealed only in cells expressing APEX-hNLRP3-GFP by CLEM (Fig. 5c, magnified in b, top).

Next, we reconstituted NLRP3 phase separation in vitro. Flag-mNG-hNLRP3 was immunoprecipitated from transfected HEK293T cells using the Flag-tag to isolate natively palmitoylated NLRP3 from cells. Upon diluting NLRP3 into the phase separation buffer with physiological pH, salt concentration and temperature, viscoelastic material was immediately formed^16,38^ (Fig. 5d). NLRP3 droplets appeared at a minimum concentration of 0.5 μM and the frequency and size of the droplets correlatively increased with the increasing concentrations of NLRP3 (Fig. 5d). The addition of a crowding agent polyethylene glycol (PEG-8000) efficiently promoted NLRP3 condensation, as expected (Fig. 5e). FRAP experiment confirmed the liquid-like properties as NLRP3 droplets recovered in 5 min after photobleaching (Fig. 5f, g). The role of palmitoylation in NLRP3 phase separation was confirmed in vitro as non-palm-NLRP3 purified from *ZDHHC7*^−/−^ cells phase-separated much weaker than palm-NLRP3 purified from the WT cells (Supplementary information, Fig. S8d). We next tested whether palmitoylation and phase separation was important for the Cryopyrin-associated periodic syndrome (CAPS)-associated NLRP3 variants. Indeed, autoactive hNLRP3 (R^262^W, D^305^N, T^350^M) mutants, each of them presumably destabilizes the inactive conformation of NLRP3,^39^ spontaneously aggregated without vesicle localization in cells and introduced C^261^S mutation impaired the auto-aggregation and auto-activation of these NLRP3 mutants (Supplementary information, Fig. S8e–g). Thus, palmitoylation is also important for phase separation and activation for CAPS-associated NLRP3 variants.

### An IDR in the FISNA domain is required for NLRP3 phase separation

Intrinsically disordered region (IDR) is generally important for protein phase separation as it mediates multiple weak interactions.^13,40^ PONDR VL-XT predicted that two regions (residues 140–152 and 159–192) in the middle of FISNA domain (residues 140–210) of hNLRP3 were highly disordered (Supplementary information, Fig. S9a, b). The FISNA domain senses low intracellular K^+^ concentration to promote NLRP3 activation, during which FISNA undergoes pronounced structural changes.^17^ Particularly, cryo-EM showed that the disordered loop 1 (residues 151–163) became ordered in the active conformation, which was involved in interaction between two neighboring NLRP3 proteins.^41^ Thus, the first predicted IDR of hNLRP3 was deleted (Δ140–152) to test its role in NLRP3 phase separation. Indeed, no condensates were found in cells expressing hNLRP3 Δ140–152 that exhibited normal palmitoylation compared to the WT (Fig. 5h, i). Consistently, there was no NLRP3 activation in hNLRP3-Δ140–152-reconstituted *NLRP3*^−/−^ THP-1 cells after LPS plus nigericin treatment that induce K^+^ efflux, neither did it respond to IMQ (Fig. 5j). Similar results were observed in mNLRP3-Δ136–148 (Supplementary information, Fig. S9c, d), suggesting that the IDR plays crucial roles in NLRP3 phase separation.

To examine the potential multivalent weak interactions mediated by this IDR, conserved hydrophobic residues V^144^F^148^I^151^ in the IDR were mutated to polar residues TYT (Supplementary information, Fig. S9e), which did not appear to affect the disorder tendency of this region (Supplementary information, Fig. S9f), Both hNLRP3 and mNLRP3 VFI/TYT mutants failed to undergo phase separation or activation (Fig. 5k, l; Supplementary information, Fig. S9g, h). Further, mutation of charged or polar residues in this IDR did not affect its aggregation and activation (Fig. 4g, h). These results suggested that hydrophobic residues in the IDR mediate multivalent weak interactions that are important for NLRP3 condensation.

### K^+^ efflux, imiquimod, cardiolipin, or palmitate induces NLRP3 phase separation in vitro and in cells

**Fig. 6:**
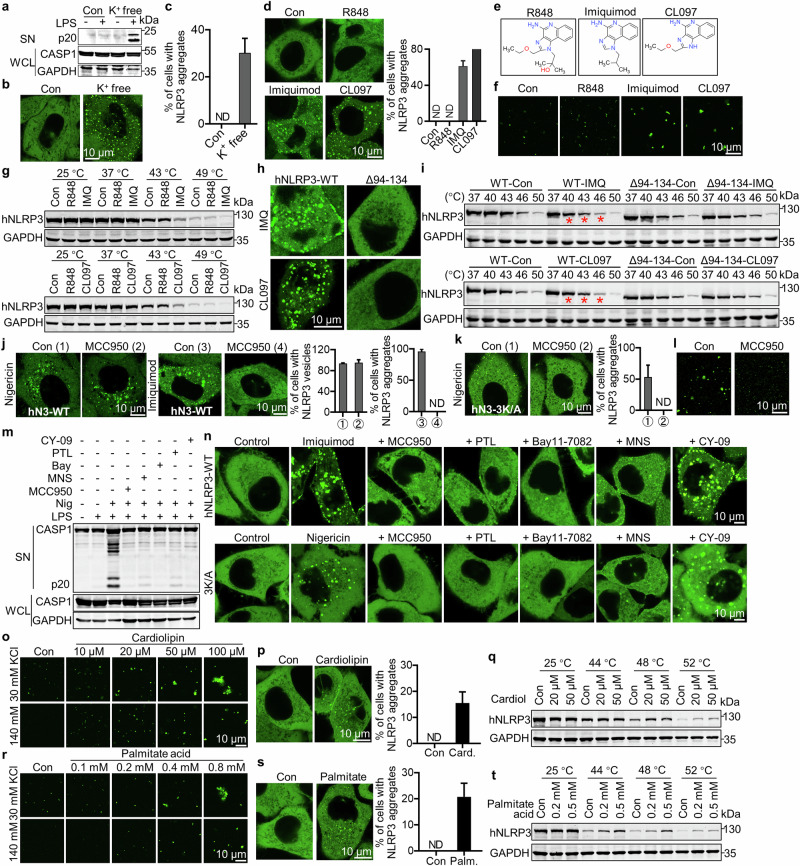
K^+^ efflux, imiquimod, cardiolipin or palmitate induces NLRP3 phase separation. **a** NLRP3 activation in THP-1 cells in control DMEM (145 mM NaCl/5 mM KCl) or K^+^ free DMEM (150 mM NaCl) for 2 h. **b**, **c** Live cell images (**b**) or percentage of cells with NLRP3 aggregates (**c**) in HeLa cells stably expressing mNG-hNLRP3 as in **a** for 5 h. **d** Live cell images (left) or percentage of cells with NLRP3 aggregates (right) in HeLa cells stably expressing mNG-hNLRP3 treated with 40 μg/mL R848, 40 μg/mL imiquimod or 40 μg/mL CL097 for 1 h. **e** The structure of R848, imiquimod, and CL097. **f** Condensation of purified mNG-hNLRP3 (1 μM) in phase-separation buffer with 60 mM KCl in the presence of 100 μg/mL R848, 100 μg/mL imiquimod, or 100 μg/mL CL097. **g** Thermostability of NLRP3 at the indicated temperatures in the presence of 1% DMSO (Con), 100 μg/mL R848, 100 μg/mL imiquimod (up), or 100 μg/mL CL097 (bottom). **h** Live cell images of HeLa cells stably expressing mNG-hNLRP3 or Δ94–134 treated with 40 μg/mL imiquimod or CL097 for 1 h. Scale bar, 10 μm. **i** Thermostability of NLRP3 or NLRP3 Δ94–134 at the indicated temperatures in the presence of 1% DMSO (Con), 100 μg/mL imiquimod (IMQ, top), or 100 μg/mL CL097 (bottom). **j**, **k** Live cell images (left) or percentage of cells with NLRP3 vesicles or aggregates (right) in HeLa cells stably expressing mNG-hNLRP3-WT treated with 8 μM nigericin or 40 μg/mL imiquimod for 1 h (**j**) or cells with mNG-hNLRP3-3K/A treated with 8 μM nigericin (**k**) in the absence or presence of 20 μM MCC950. **l** In vitro NLRP3 (1 μM) LLPS in a phase-separation buffer in the presence of 1 mM MCC950. **m** Immunoblotting analysis of NLRP3 activation in THP-1 cells pretreated with 2 μM MCC950, 10 μM parthenolide (PTL), 10 μM Bay11-7082, 10 μM MNS or 20 μM CY-09 for 30 min, and then treated with 1 μg/mL LPS for 3 h, followed by 4 μM nigericin treatment for another 1 h. **n** Live cell images of HeLa cells stably expressing mNG-hNLRP3 (top) or mNG-hNLRP3 -3K/A (bottom) untreated (Control), or pretreated with DMSO, 2 μM MCC950, 10 μM PTL, 10 μM Bay11-7082, 10 μM MNS or 20 μM CY-09 for 30 min, followed by 40 μg/mL imiquimod (top) or 8 μM nigericin treatment (bottom) for 1 h. Scale bar, 10 μm. **o** In vitro NLRP3 LLPS as in **l** with the indicated concentrations of KCl and cardiolipin. **p** Images (left) or percentage of cells with NLRP3 aggregates (right) in HeLa cells stably expressing mNG-hNLRP3 treated with 120 μM cardiolipin for 5 h before live cell imaging. **q** Thermostability of NLRP3 at the indicated temperatures in the presence of 5% methanol (Con), 20 μM or 50 μM cardiolipin. **r** In vitro NLRP3 LLPS as in **l** with indicated concentrations of KCl and palmitate acid. **s** Images (left) or percentage of cells with NLRP3 aggregates (right) in HeLa cells stably expressing mNG-hNLRP3 treated with 100 μM palmitate for 3 h before live cell imaging. **t** Thermostability of NLRP3 at the indicated temperatures in the presence of 3% methanol and 1% chloroform (Con), 200 μM or 500 μM palmitate acid.

Previous studies showed that K^+^ efflux is induced by almost all NLRP3-activating stimuli, which also modifies the NLRP3 structure.^17^ Macromolecular phase separation is highly sensitive to ionic strength,^13^ and we therefore tested if K^+^ levels would regulate NLRP3 phase separation. Indeed, NLRP3 did not aggregate in a buffer with the ion level close to the physiological one (140 mM KCl or NaCl), lower salt (K^+^ or Na^+^) concentrations readily induced NLRP3 phase separation (Fig. 5m; Supplementary information, Fig. S9i). Consistent with previous results in cells (Fig. 5j), only the WT hNLRP3, but not the hNLRP3 Δ140–152, phase separated (Supplementary information, Fig. S9j). Reduced K^+^ level in the culture medium potently induced NLRP3 aggregation and activation in cells (Fig. 6a–c).

NLRP3 is also activated by IMQ and derivatives independent of K^+^ efflux via targeting mitochondria to induce ROS and thiol oxidation.^42,43^ Interestingly, we found that IMQ, CL097, but not R848, induced NLRP3 phase separation in vitro and in cells (Fig. 6d–f). Since they induced NLRP3 condensation in vitro, the role of ROS in inducing NLRP3 condensation by these two molecules was most likely excluded. These results indicated that in addition to ROS-mediated oxidation that affects protein conformation in cells,^42^ IMQ and CL097 induced NLRP3 aggregation and activation probably via IMQ/CL097-caused NLRP3 conformational change, as previous study suggested.^17^ Consistently, cellular thermal shift assay (CETSA) revealed that both IMQ and CL097, but not R848, changed NLRP3 stability (Fig. 6g), most likely by binding NLRP3 directly as previous work demonstrated.^17^ Since the N-terminal region (94–134 AA for hNLRP3, the same sequence as 92–132 AA in the literature^17^) is important for IMQ-induced NLRP3 activation, we tested the interaction and response of hNLRP3 (∆94–134) to IMQ and CL097. We found that deletion of this fragment completely abolished IMQ/CL097-induced NLRP3 phase separation (Fig. 6h) and lost the interaction with IMQ and CL097 as indicated by unchanged thermostability in CETSA (Fig. 6i), indicating that IMQ and CL097 directly bind to NLRP3 by this region. Moreover, the NLRP3 specific inhibitor MCC950 suppressed NLRP3-3K/A and NLRP3 aggregation, but not nigericin-induced NLRP3 vesicle recruitment in cells (Fig. 6j, k). In vitro, MCC950 also inhibited low K^+^-induced NLRP3 LLPS (Fig. 6l). These results agreed with previous studies showing that MCC950 suppressed NLRP3 aggregation^44^ but not vesicle recruitment in cells.^19^ Similarly, ADP or dATP suppressed low K^+^-induced NLRP3 LLPS (Supplementary information, Fig. S10a). Since MCC950 directly binds the NACHT and LRR domains of NLRP3 to prevent its conformational change,^44–46^ inactive NLRP3 can be similarly stabilized by dATP^19^ or ADP.^39^ Consistently, MCC950 binding unlikely affects the exposure of the polybasic region, which is localized upstream of NACHT and LRR domains, thus keeping NLRP3 vesicle recruitment unchanged in cells. These results confirmed that the conformational change of NLRP3 is important for its phase separation.

We further tested several other compounds known to block the ATPase activity of NLRP3, such as the vinyl sulfone Bay11-7082, the sesquiterpene lactone parthenolide (PTL), 3,4-methylenedioxy-b-nitrostyrene (MNS), and the CFTR(inh)-172 (C172) analog CY-09. MNS binds to NACHT and LRR domains of NLRP3, similar to MCC950, to block ATPase activity. It also modifies cysteine in NLRP3 via its nitrovinyl group.^47^ Bay11-7082 or PTL inhibits NLRP3 ATPase through an irreversible covalent cysteine alkylation without characterized binding sites on NLRP3.^48–50^ CY-09 binds to the Walker A motif of NLRP3 to abolish its ATP binding and ATPase activity.^51^ We found that similar to MCC950, MNS, Bay11-7082, or PTL potently inhibited both LLPS and subsequent activation of NLRP3. Surprisingly, CY-09 only blocks NLRP3 inflammasome activation (Fig. 6m) but not NLRP3 phase separation in cells or in vitro (Fig. 6n; Supplementary information, Fig. S10b), consistent with previous results that CY-09 competes with ATP for binding but not locks NLRP3 in an inactive conformation as MCC950 or MNS does or covalently inactivates NLRP3 via irreversible cysteine modification and fixed NLRP3 conformation as MNS, Bay11-7082, or PTL does. These results strongly indicated that LLPS of NLRP3 precedes its ATPase activity-required ATP-dependent opening of NLRP3 and the release of the PYD domain.

Previous work reported that the mitochondrial lipid cardiolipin directly binds and activates NLRP3.^52^ We found that cardiolipin potently induced NLRP3 aggregation in vitro and in cells (Fig. 6o, p). CETSA results also indicated a direct binding of NLRP3 by cardiolipin (Fig. 6q). Thus, similar to IMQ or CL097, cardiolipin may also bind and cause NLRP3 conformational change to induce NLRP3 condensation and downstream activation.

Recent studies have reported that palmitate, one of the most abundant saturated fatty acids in plasma elevated by high-fat diet, activates NLRP3.^53^ Since cardiolipin and palmitate share similar fatty acyl chains with 16-carbon (saturated, in palmitate) or 18-carbon (unsaturated, in cardiolipin) and both activate NLRP3, we went to test if palmitate works on NLRP3 similarly to cardiolipin. We found that palmitate induced NLRP3 aggregation in vitro and in cells (Fig. 6r, s), probably by interacting with and causing NLRP3 conformational change as suggested by CETSA (Fig. 6t). Thus, the intracellular metabolite palmitate may regulate NLRP3 inflammasome activation by directly modulating the phase separation of NLRP3. Moreover, since cardiolipin and palmitate have been shown to induce K^+^ efflux-dependent NLRP3 activation in cells, we reasoned that both K^+^ efflux- and NLRP3 interaction-caused conformational changes may be involved in cardiolipin- or palmitate-induced NLRP3 activation. Indeed, we found that both cardiolipin and palmitate induced NLRP3 aggregation in vitro more dramatically in 30 mM potassium, at which level NLRP3 is maximally activated in cells,^42^ compared to that in 140 mM potassium, a concentration similar to its intracellular level (Fig. 6o, r), indicating that conformational changes caused by both K^+^-efflux and cardiolipin- or palmitate-binding have a synergistic NLRP3-activating effect.

### Amphiphilic molecules induce NLRP3 phase separation and activation

**Fig. 7:**
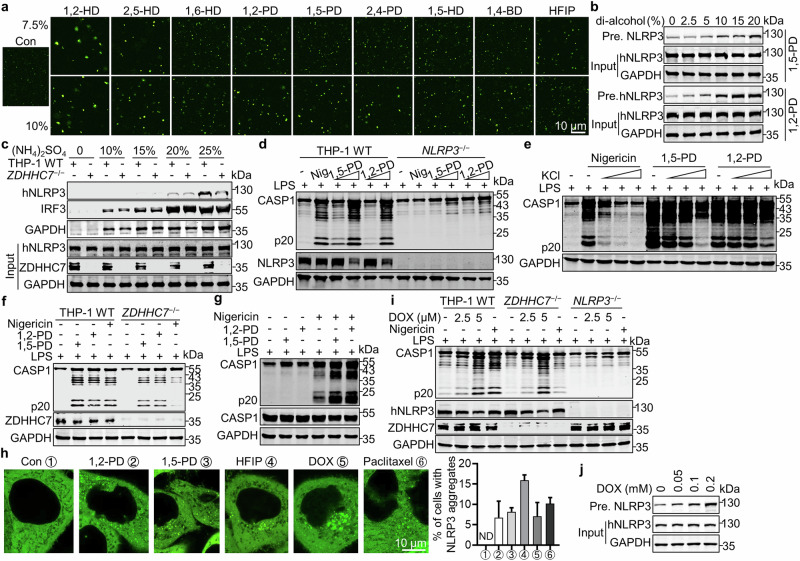
Amphiphilic molecules induce NLRP3 phase separation and activation. **a** In vitro NLRP3 LLPS as in Fig. 6l with the indicated amphiphilic molecules at different concentrations (7.5% or 10%). Scale bar, 10 μm. **b** The solubility of NLRP3 in whole cell lysates of HEK293T cells expressing Flag-hNLRP3 in the presence of indicated concentration of amphiphilic di-alcohols including 1,5-PD and 1,2-PD. Precipitated NLRP3 (Pre. NLRP3) was detected by immunoblotting. **c** The solubility of NLRP3 in cell lysates of WT and *ZDHHC7*^−/−^ THP-1 cells in the presence of the indicated concentrations of ammonium sulfate (g/100 mL whole cell lysate) was analyzed. Experiment was conducted as in (**b**). **d** NLRP3 activation in WT or *NLRP3*^–/–^ THP-1 cells treated with 1 μg/mL LPS for 3 h, followed by 4 μM nigericin for another 1 h, or cells pretreated with 1 μg/mL LPS for 30 min, followed by 1,5-PD (1.2%, 2.5%) or 1,2-PD (0.6%, 1.2%) treatment for another 3 h. **e** NLRP3 activation in THP-1 cells treated by 4 μM nigericin, 2.5% 1,5-PD or 1.2% 1,2-PD in the presence of different concentrations of KCl (5, 10, 20 mM). **f** NLRP3 activation in the WT or *ZDHHC7*^–/–^ THP-1 cells treated by nigericin (4 μM), 1,5-PD (2.5%) or 1,2-PD (1.2%) as in (**d**). **g** NLRP3 activation in THP-1 cells treated by nigericin in the presence of 1,5-PD, 1,2-PD or not. Cells were pretreated with 1 μg/mL LPS for 2 h, followed by 0.6% 1,5-PD or 0.3% 1,2-PD treatment for 1 h, and 2 μM nigericin treatment for another 1 h. **h** Live cell images (left) or percentage of cells with NLRP3 aggregates (right) in HeLa cells stably expressing mNG-hNLRP3 with the indicated treatment, 0.5% 1,2-PD or 0.5% 1,5-PD for 4 h, 0.1% HFIP or 20 μM doxorubicin for 3 h, or 5 μM paclitaxel for 4 h. **i** NLRP3 activation in WT, *ZDHHC7*^–/–^, or *NLRP3*^–/–^ THP-1 cells pretreated with 0.2 μg/mL LPS for 3 h and 2 μM nigericin for another 1 h, or pretreated with 0.2 μg/mL LPS for 30 min and doxorubicin (DOX, 2.5 μM, 5 μM) for another 8 h. **j** NLRP3 solubility in cell lysate from HEK293T cells expressing Flag-hNLRP3 in the presence of indicated concentration of doxorubicin. Precipitated NLRP3 (Pre. NLRP3) was detected by immunoblotting.

Aliphatic alcohols such as 1,6-hexanediol (1,6-HD) is commonly used to disrupt biomolecular condensates, presumably by reducing hydrophobic interactions, whereas the isomeric 2,5-hexanediol (2,5-HD) is a negative control with much less disruptive activity.^54^ Surprisingly, when 1,6-HD or 2,5-HD was used to disrupt NLRP3 condensates in vitro, both of them instead promoted NLRP3 phase separation in a dose-dependent way (Fig. 7a), whereas 1,6-HD, but not 2,5-HD, efficiently disrupted STING-overexpression-induced STING phase-separators as previously reported^37^ (Supplementary information, Fig. S10c). To confirm this result, NLRP3 in a buffer with 140 mM KCl was exposed to other amphiphilic diols including 1,4-butanediol (1,4-BD), 1,2-pentanediol (1,2-PD), 1,5-pentanediol (1,5-PD), 2,4-pentanediol (2,4-PD), 1,2-hexanediol (1,2-HD), and 1,5-hexanediol (1,5-HD). It was found that all tested di-alcohols promoted NLRP3 condensation in vitro to some degree (Fig. 7a). Previous studies found that both 1,6-HD and 2,5-HD remove water molecules around chromatin to locally condense chromatin and initiate its condensation.^55^ We reasoned that amphiphilic di-alcohols may similarly decrease NLRP3 solubility in cytosol and promote its phase separation. Indeed, we found that all tested amphiphilic di-alcohols obviously decreased the solubility of NLRP3 in a concentration-dependent manner (Fig. 7b; Supplementary information, Fig. S10d). Consistently, compared to GAPDH and IRF3 that were readily precipitated with 10% (NH_4_)_2_SO_4_, NLRP3 started to precipitate with 20% (NH_4_)_2_SO_4_, indicating a higher solubility of NLRP3 (Fig. 7c). When used to treat THP-1 cells, 1,6-HD, 1,5-PD, and 1,2-PD showed the strongest NLRP3-activating activity followed by 1,4-BD and 1,2-HD. 1,5-HD displayed no apparent activity (Fig. 7d; Supplementary information, Fig. S10e, f), most likely due to its high toxicity to cells. Consistently, treatment with MCC950 or CY-09 suppressed NLRP3 activation induced by 1,5-PD and 1,2-PD (Supplementary information, Fig. S10g), indicating again that LLPS of NLRP3 precedes its ATPase activity-required opening of NLRP3. Interestingly, preventing potassium efflux with 20 mM extracellular KCl partially inhibited diol-induced NLRP3 activation, in contrast to nigericin-treated cells that showed almost no NLRP3 activation with 5 mM extracellular KCl (Fig. 7e), indicating that amphiphilic diol-induced NLRP3 activation is somehow resistant to K^+^ efflux. Previous studies found that amphiphilic alcohol forms hydrophobic interactions with hexafluoro isopropanol (HFIP) and causes the latter to aggregate,^56^ Consistently, HFIP also induced NLRP3 aggregation and activation. Inspired by these results, we hypothesized that palmitoylation-conferred hydrophobicity to NLRP3 may also decrease its solubility, thus promoting phase separation. To test this, we compared the solubility of palmitoylated and non-palmitoylated NLRP3 and found that palmitoylation did decrease NLRP3 solubility (Fig. 7c; Supplementary information, Fig. S10h). Moreover, amphiphilic di-alcohols decreased the solubility of both palmitoylated and non-palmitoylated NLRP3 (Supplementary information, Fig. S10i). Based on these results, we speculated that amphiphilic di-alcohols may bypass ZDHHC7-mediated NLRP3 palmitoylation and directly induce NLRP3 phase separation and activation. Indeed, unlike nigericin that is heavily dependent on ZDHHC7, amphiphilic di-alcohols activated NLRP3 in *ZDHHC7*^−/−^ THP-1 cells almost as well as in the WT cells (Fig. 7f). Importantly, amphiphilic alcohols enormously enhanced nigericin-induced NLRP3 activation (Fig. 7g), indicating a great synergistic effect.

Chemotherapy is one of the most commonly used approaches to treat tumors but often leads to serious inflammation. Previous studies showed that doxorubicin and paclitaxel activated NLRP3.^57^ Both drugs are hydrophobic or near amphiphilic.^58^ We found that either doxorubicin or paclitaxel induced NLRP3 aggregation in vitro and in cells (Fig. 7h; Supplementary information, Fig. S10j), indicating that amphiphilic molecules directly promote the phase separation of NLRP3 and activation. Similar to amphiphilic di-alcohols, both doxorubicin and paclitaxel decreased solubility of NLRP3 and activated NLRP3 in *ZDHHC7*^−/−^ THP-1 cells (Fig. 7i, j; Supplementary information, Fig. S10k, l). Notably, similar to NLRP3-interacting molecules including ATP, IMQ, cardiolipin and palmitate, all these tested amphiphilic molecules induced NLRP3 phase separation without NLRP3 vesicle recruitment (Figs. 6p, s, and 7h; Supplementary information, Fig. S7q), in sharp contrast to nigericin-treated HeLa cells showing prominent NLRP3 vesicles.

## Discussion

Dysregulated NLRP3 activation is involved in many inflammatory, metabolic, and neurodegenerative diseases. NLRP3 is activated by diverse unrelated stimuli including infections, DAMPs, cellular and environmental factors, all inducing cellular stress.^21^ It is also considered an intracellular safeguard to sense altered metabolites such as fatty acids and cholesterol. How NLRP3 senses cellular stress or danger signals and gets activated is unknown. Although hypotheses propose various unidentified or yet-to-be-confirmed 2^nd^ messenger to activate NLRP3, none is suitable for all stimuli. Here we reported that signal-dependent NLRP3 phase separation is uniformly required for its activation serving as the long-debated upstream event. We showed that all NLRP3 activators in this study induce NLRP3 phase separation and activation in cells. In fact, one of the best characterized features of phase separation is its responsiveness to various environmental alterations or cell stress, consistent with multi-signal-activated NLRP3. We proposed at least three modes of action by signal-induced NLRP3 phase separation. Intracellular changes, including ion efflux or influx, trigger NLRP3 phase separation by inducing its conformational change; NLRP3-binding molecules like cardiolipin, palmitate, and IMQ directly cause NLRP3 conformational change and phase separation; and amphiphilic molecules initiate NLRP3 phase separation by reducing NLRP3 solubility in a ZDHHC7-independent manner. The first two types of signal-induced NLRP3 phase separation are dependent on ZDHHC7-mediated palmitoylation. Therefore, our findings probably provide the simplest and most direct mechanistic basis for NLRP3 activation. Our results also raise a caveat for the use of amphiphilic molecules/drugs known to be most suitable for drug delivery, as they tend to establish an intracellular environment in favor of NLRP3 aggregation.

Previous work reported that ZDHHC7-palmitoylated STAT3^59^ or ZDHHC5-palmitoylated NOD1/2^60^ is important for their membrane binding and immune signaling. While these results, consistent with other work, support the importance of protein palmitoylation for its membrane localization, our results for the first time reported protein palmitoylation regulated its phase separation. We demonstrated that ZDHHC7-mediated continuous NLRP3 palmitoylation was a prerequisite for NLRP3 phase separation and activation in response to most known activators while ABHD13 functions in the opposite way. Since palmitoylation provides hydrophobicity and it is well known that hydrophobic interaction is important for macromolecular phase separation, ZDHHC7-mediated NLRP3 palmitoylation may be required for hydrophobic interactions, in addition to the hydrophobic residues-mediated multivalent weak interactions in the IDR of NLRP3. We also showed that NLRP3 palmitoylation decreased the solubility of NLRP3 in cytosol to promote its phase separation, a phenomenon confirmed by amphiphilic di-alcohols-induced NLRP3 phase separation and activation. Accordingly, this study may offer a potential therapeutic strategy for the treatment of NLRP3-related diseases as pharmacological inhibition of protein palmitoylation improved mouse survival by endotoxic shock and small peptides harboring NLRP3 palmitoylation sites suppressed NLRP3 activation.

One recent study reported NLRP3 palmitoylation by ZDHHC5 at the priming stage.^29^ There are several differences between ZDHHC5- and ZDHHC7-mediated NLRP3 palmitoylation. Firstly, ZDHHC5 mediates hNLRP3 palmitoylation at sites C^837/838^, while ZDHHC7 does at sites C^130/261^ (human) or C^126^ (mouse). Secondly, ZDHHC7 constitutively palmitoylates and licenses NLRP3 activation, whereas ZDHHC5 palmitoylates NLRP3 only after LPS treatment. Finally, ZDHHC5 palmitoylates NLRP3 to promote NEK7–NLRP3 interaction, whereas ZDHHC7 palmitoylates NLRP3 to enhance NLRP3 phase separation. Moreover, after NLRP3 activation, ZDHHC12 is induced to palmitoylate NLRP3 at C^844^ and promote its degradation in lysosomes, thus negatively regulates NLRP3 inflammasome.^26^ ZDHHC12 was not enriched in this CRISPR-Cas9 screening, probably due to THP-1 cells used by us expressing an autoactive mutant of NLRP3 (R^262^W) that nullifies the negative regulation by ZDHHC12. During the revision of this manuscript, ZDHHC1 has been reported to palmitoylate hNLRP3 at C^958^ and C^130^ in the priming and activation stages and is important for NLRP3 membrane trafficking,^61^ suggesting that ZDHHC1 may cooperate with ZDHHC7 for adequate NLRP3 palmitoylation to ensure its activation. Thus, NLRP3 palmitoylation by different ZDHHCs occurs in different cell states with distinct effects.

## Materials and methods

### Reagents

RPMI-1640, DMEM, Opti-MEM, and fetal bovine serum (FBS) were purchased from Gibco. Cerulenin (HY-A0210), Z-VAD-FMK (HY-16658B), CL097 (HY-128799), MNS (HY-78263), Bay11-7082 (HY-13453), Parthenolide (HY-N0141), CY-09 (HY-103666) and Imiquimod (HY-B0180A) were obtained from MCE. 2-Bromohexadecanoic acid (21604-1 g), FTI-276 (344550-250UG), GGTI-2133 (G5294-1MG), palmitic acid (P0500-10G), LPS (L2880-100MG), tris((1-benzyl-1H-1,2,3-triazol-4-yl) methyl)amine (TBTA; 678937-50MG), N-ethylmaleimide (NEM; E3876-5G), palmostatin B (5087380001), S-methyl methanethio-sulfonate (MMTS; 64306-1 ML), hydroxylamine solution (467804-50 ML), ATP (A2383-10G), nigericin (N7143), Flag beads (A220), Kolliphor® EL (C5135-500G), and MSU (U2875) were acquired from Sigma-Aldrich. BODIPY^TM^ 665/676 (B3932) was purchased from Invitrogen. Pam2CSK4 (tlrl-pm2s-1), R848 (tlrl-r848-5), CRX527 (tlrl-crx527), and poly(dA:dT) (tlrl-patn) were purchased from Invivogen. EZ-Link^TM^ BMCC-Biotin (21900), Pierce^TM^ tris-(2-carboxyethyl)-phosphine hydrochloride (TCEP-HCl; 20490), and streptavidin beads (20359) were acquired from Thermo Fisher. Paclitaxel (S1150) and Doxorubicin (S1208) were obtained from Selleck. Palmitate (BD379945), 1,2-PD (BD53502-5G), 2,4-PD (BD67269), 1,4-BD (BD54499-25G) and HFIP (BD35431-10G) were obtained from Bidepharm. 1,2-HD (H862045-1G), 2,5-HD (H823950-5mL), 1,6-HD (H810887-100G) and 1,5-HD (H921565-1G) were obtained from Macklin. Cardiolipin (C130308-5mg) was obtained from Aladdin. 1,5-PD (P-00990) was obtained from Heowns. Puromycin (13844), 17-ODYA (90270), biotin-PEG3-azide (23419), and N-(6-[biotinamido]hexyl)-3’- (2’-pyridyldithio) propionamide (biotin-HPDP; 16459) were obtained from Cayman. *Salmonella* Typhimurium (CMCC 50222) was a gift from Dr. Rongbin Zhou; VACV (Western Reserve-Vvt7 strain) was a gift from Dr. Bernard Moss.

### Antibodies

Flag (F3165) and HA (H9658) antibodies were obtained from Sigma-Aldrich. NLRP3 (AG-20B-0014-C100) antibody was acquired from AdipoGen. Human caspase-1 (22915-1-AP) antibody was purchased from Proteintech. GFP (sc-8334) and glyceraldehyde-3-phosphate dehydrogenase (GAPDH; sc-25778) antibodies were acquired from Santa Cruz. mCherry (BE2026) antibody was purchased from Easybio. GM130 (ab52649), Rab5 (ab218624), TGN46 (ab50595), and NLRP3 (ab270449) antibodies were obtained from Abcam. Lysosomal-associated membrane protein (LAMP)1 (3243) and Rab7 (9367) antibodies were procured from Cell Signaling Technology. Streptavidin Protein, DyLight™ 800 (21851) antibody and ZDHHC7 (PA5-116795) antibody was purchased from Thermo Fisher. Some antibodies were made in our laboratory: cDNAs of human ABHD13 (225–337 AA), human GSDMD (276–484 AA), human IL-1β (117–269 AA), mouse caspase-1 (121–296 AA), mouse IL-1β (118–269 AA), and human ASC (1–195 AA) were cloned into the pET-21b vector (Novagen) and expressed in *Escherichia coli* BL21 (DE3). The recombinant proteins were purified by Ni-NTA affinity chromatography, then injected into mice to produce antiserum.

### Mice

*Zdhhc7*^−/−^ (S-KO-00236) and *Abhd13*^−/−^ (S-KO-12879) mice were generated by and purchased from Cyagen Biosciences. All mice were bred and maintained under specific pathogen-free conditions in the Laboratory Animal Center of Peking University. Sex and age-matched mice were analyzed. Experiments were performed in accordance with the National Institutes of Health Guide for Care and Use of Laboratory Animals (LSC-JiangZF-1), with the approval of the Peking University Laboratory Animal Center, Beijing.

### Cells

THP-1, HEK293T, B16, and HeLa cell lines were obtained from ATCC. The iBMDM cell line was a gift from Dr. Katherine Fitzgerald (University of Massachusetts Medical School). THP-1 cells were cultured in RPMI-1640 medium supplemented with 10% FBS, 5 μg/mL of penicillin, and 10 μg/mL of streptomycin. HEK293T, B16, iBMDM, and HeLa cells were cultured in DMEM supplemented with 10% FBS, 5 μg/mL of penicillin, and 10 μg/mL of streptomycin.

### Virus amplification and infection

To produce lentivirus, lentiviral vectors and two viral packaging plasmids (pMD2.G and psPAX2) were cotransfected into HEK293T cells. Supernatants containing lentivirus were harvested 48 h after transfection. Cells were cocultured with lentivirus-containing supernatants for 12 h, and then supernatants were replaced with fresh medium. After cells had been cultured in fresh medium for 48 h, they were subjected to fluorescence-activated cell sorting or antibiotic selection.

### Genome-wide CRISPR-Cas9 screening

A pooled human sgRNA plasmid library targeting 19,114 human genes was purchased from Addgene (Brunello, #73178).^62^ Lentivirus production and infection were performed as described above.^63^ In the screening assay, THP-1 Cas9 cells were transduced with the sgRNA lentiviral library at a multiplicity of infection (MOI) of 0.2 and with 1000× coverage. After 5–6 days of selection with puromycin (1 μg/mL), puromycin-resistant cells were divided into two groups. One group of cells was continuously cultured, with fresh medium provided at 2-day intervals. The other group of cells was pretreated with 10 μM MCC950 for 30 min, then treated with 100 ng/mL LPS for 2 h. Subsequently, the cells were provided fresh medium and treated with 100 ng/mL LPS at 2-day intervals. After 21 days, LPS-resistant cells were collected, and genomic DNA was extracted using the phenol/chloroform method. Next, polymerase chain reaction (PCR) amplification was performed following the reagent protocol (TransTaq DNA Polymerase High Fidelity) using the primers lentiGP-1_F (AATGGACTATCA TATGCTTACCGTAACTTGAAAGTATTTCG) and lentiGP-3_R (ATGAATACTGCCATT TG TCTCAAGATCTAGTTACGC). PCR products were purified following the reagent manufacturer’s instructions (Thermo), then subjected to high-throughput deep sequencing analysis.

### Generation of gene-deficient cells and NLRP3-R^262^W (heterozygous) cells

Lentiviruses containing sgRNA and specific genes were used as described above. *ZDHHC7*^−/−^ THP-1 cells, THP1-NLRP3-R^262^W (heterozygous), *ZDHHC7*^−/−^ HeLa cells, *ABHD13*^−/−^ THP-1 cells, *ABHD13*^−/−^ HeLa cells, and *Zdhhc7*^−/−^ iBMDM cells were generated via CRISPR-Cas9 system (*ZDHHC7* sgRNA: CCGAGGCTGACGTGGCTGAC, *Zdhhc7* sgRNA: CCGAGACTG ACATGGCAGAC, *ABHD13* sgRNA: ATGCGTTACCTTCCTTTATG, *NLRP3* sgRNA: CAAG GCTCACCTCTCGACAG). Genotyping of targeted genes in the indicated cells is listed in the Supplementary information, Table S1.

### Immunoblotting

Supernatants were precipitated with water/chloroform/methanol (v/v 4:1:4), then centrifuged at 20,000× *g* for 15 min. The resulting protein pellets were washed once with 700 μL of prechilled methanol, then centrifuged at 20,000× *g* for 10 min. Next, the protein pellets were briefly air-dried, resuspended in sodium dodecyl sulfate (SDS) loading buffer, and heated at 95 °C for 10 min; the samples were separated by SDS-PAGE and blotted following a standard protocol.

Cells were lysed in immunoprecipitation lysis buffer (0.5% Triton X-100, 20 mM 4-(2-hydroxyethyl)-1-piperazineethanesulfonic acid (HEPES, pH 7.4), 150 mM NaCl, 12.5 mM β-glycerophosphate, 1.5 mM MgCl_2_, 2 mM EGTA, 10 mM NaF, 2 mM dithiothreitol, 1 mM Na_3_VO_4_, and 1 mM phenylmethylsulfonyl fluoride (PMSF) for 30 min at 4 °C, then centrifuged at 20,000× *g* for 15 min at 4 °C to collect cell lysate. Subsequently, the lysates were mixed with SDS loading buffer and heated at 95 °C for 6 min, then subjected to immunoblotting.

### Co-immunoprecipitation

NLRP3 and ABHD13 or ZDHHC7 were cotransfected into HEK293T cells for 24 h. The cells were washed with cold phosphate-buffered saline (PBS) and resuspended in 400 μL of immunoprecipitation lysis buffer for 60 min at 4 °C, and then centrifuged at 20,000× *g* for 15 min at 4 °C to collect cell lysates. For each sample, 30 μL was used as a loading control and 370 μL was incubated with anti-GFP beads or anti-Flag beads overnight at 4 °C. Beads were washed three times with PBS and eluted with 40 μL of SDS buffer (100 mM Tris-HCl, pH 6.8, 4% SDS, 20% glycerol, 0.2 M dithiothreitol, and 0.2% bromophenol blue) via heating at 95 °C for 10 min. Subsequently, the samples were used for immunoblotting.

### Inflammasome activation assay

THP-1 cells, iBMDMs, BMDMs, or peritoneal macrophages were seeded in 12-well plates with Opti-MEM. The cells were then primed with LPS (1 μg/mL), Pam2CSK4 (10 ng/mL), or CRX527 (2 μg/mL) for 3 h, followed by exposure to different inflammasome ligands. For NLRP3 inflammasome activation, cells were treated with ATP (5 mM) for 60 min, nigericin (4 μM) for 60 min, SiO_2_ (500 μg/mL) for 4 h, imiquimod (30 μg/mL) for 90 min, or VACV (MOI = 0.1) for 4 h.^44^ For amphiphilic diols activation of hNLRP3 in THP-1 cells, the cells were then primed with LPS (1 μg/mL) for 30 min, followed by exposure to different amphiphilic diols including 1,6-HD (1.2%, 2.5%), 2,5-HD (1.2%, 2.5%),1,4-BD (1.2%, 2.5%), 1,2-PD (0.6%, 1.2%), 1,5-PD (1.2%, 2.5%), 2,4-PD (0.6%, 1.2%), 1,2-HD (0.3%, 0.6%), HFIP (0.15%, 0.3%), and 1,5-HD (0.3%, 0.6%) for 3–4 h. For autoactive hNLRP3 activation, cells were treated with LPS (1 μg/mL) or Pam2CSK4 (10 ng/mL) for 1 h. For AIM2 inflammasome activation, cells were transfected with poly(dA:dT) (1 μg/mL) for 4 h.^1,2,64,65^ For NLRC4 inflammasome activation, cells were treated with *Salmonella* Typhimurium (MOI = 0.5) for 60 min.^1,2,66^ For PI staining, PI (3.33 μg/mL) was added before inflammasome ligands. Cells and medium (supernatant) were both collected for immunoblotting as described above.

### LDH assay

LDH release was evaluated by CyQUAN LDH assay kit, in accordance with the manufacturer’s instructions (Invitrogen).

### Cell viability measurement

THP-1 cell viability was measured by 3-(4, 5-dimethylthiazol-2-yl)-2, 5-diphenyltetrazolium bromide (MTT) assay (V13154, Invitrogen), in accordance with the manufacturer’s instructions.

### Immunofluorescence microscopy

HeLa cells or iBMDMs were seeded on glass coverslips overnight. THP-1 cells were seeded on glass coverslips incubated with polylysine before treatment. The glass coverslips were incubated with polylysine at 37 °C for 30 min, and then washed with PBS. THP-1 cells were seeded on them for 30 min to keep cells adherent. For NLRP3 activation in HeLa cells, cells were treated with nigericin (8 μM) for 60 min; for NLRP3 activation in iBMDM or THP-1 cells, cells were treated with 1 μg/mL LPS for 3 h and 4 μM or 6 μM nigericin for 1 h, then fixed in 4% paraformaldehyde for 30 min. Cells were permeabilized with 0.5% Triton X-100 for 15 min, blocked with 5% bovine serum albumin for 30 min, then incubated with primary antibodies (anti-Flag: 1:100; anti-HA: 1:100; anti-NLRP3: 1:200) in 5% bovine serum albumin overnight at 4 °C. Cells were washed three times with PBS, then incubated with secondary antibody (1:100; Invitrogen) for 1 h. Confocal images were acquired with a Nikon N1R confocal microscope under a 60× objective (numerical aperture 1.45). For each sample, ≥ 5 fields were chosen to analyze the proportion of cells containing NLRP3 puncta after nigericin treatment.

For HeLa cells stably expressing mNG-hNLRP3, live cell imaging was used after nigericin (8 μM) treatment for 1 h, imiquimod (40 μg/mL) for 1 h, R848 (40 μg/mL) for 1 h, 0.1% HFIP for 3 h, 400 μM palmitate for 3 h, 5 μM paclitaxel for 4 h, 20 μM doxorubicin for 3 h, 0.5% 1,2-PD for 4 h or 0.5% 1,5-PD for 4 h. For time-lapse photography, live cell imaging was taken at indicated time after nigericin (8 μM) treatment. For inhibitors, cells were pretreated with 50 μM 2-BP^67,68^ overnight, 10 μM cerulenin,^67,68^ 20 nM FTI-276, 10 μM GGTI-2133 or 20 μM MCC950^45,46,69^ for 1 h, then stimulated with 8 μM nigericin for another 1 h. For NLRP3 ATPase inhibitors, cells were pretreated with 2 μM MCC950, 10 μM parthenolide, 10 μM Bay11-7082, 10 μM MNS, or 20 μM CY-09 for 30 min, then stimulated with 8 μM nigericin or 40 μg/mL imiquimod for another 1 h.

For BODIPY staining, nigericin treated HeLa cells or HEK393T cells were collected in ice-cold PBS and sheared through a needle to get the whole cell lysates. 10 μg/mL BODIPY was added to the lysates and incubated for 30 min on ice before imaging.

### TEM

HeLa cells stably expressing APEX-hNLRP3-EGFP-A^206^K or APEX-hNLRP3-4K/A-EGFP -A^206^K were plated on the coverslips for 24 h. Cells were fixed and stained according to the procedure before.^37^ Sections were cut by an ultramicrotome (Leica Microsystem, FC7) for 120-kV TEM (FEI, Tecnai G2 Spirit) examination.

### FRAP

Cellular FRAP experiments were performed on a Nikon AXR confocal microscope at 37 °C. HeLa cells were cultured on 35-mm glass-bottom dishes for 24 h and then treated with nigericin for different time. hNLRP3 condensates were fully or partially photobleached with 50% laser power for 1–3 s using a 488 nm laser. Time-lapse images were taken every 10 s after bleaching. FRAP data were analyzed with Image J.

For the in vitro FRAP assay, experiments were performed on a Nikon A1R+ confocal microscope and Nikon AXR confocal microscope at RT. Condensates were photobleached with 40% laser power for 1–2 s using 488 nm lasers. Time-lapse images were taken every 10 s after bleaching. FRAP data were analyzed by Image J.

### Expression and purification of hNLRP3 protein

Full-length human NLRP3 was purified from HEK293T WT or *ZDHHC7*^−/−^cells transfected with plasmid expressing Flag-mNG-hNLRP3. Cell pellets were washed with PBS once and resuspended in a buffer containing 50 mM HEPES, 500 mM NaCl, 5 mM MgCl_2_, pH 7.5, supplemented with protease inhibitor cocktail and 1 mM TCEP before usage. Cells were sonicated (3 s on, 6 s off, 3 min total on, 40% power), centrifuged at 14,000 RPM for 30 min, the supernatant was filtered through a 0.45 μm syringe filter and then was used for affinity chromatography with anti-FLAG M2 affinity gel by overnight. The beads were washed with 10 column volumes of the lysis buffer and the protein was eluted with 100 μg/mL 3*×* FLAG peptide (HY-P0319, MCE). And the hNLRP3 protein was concentrated and replaced with a buffer containing 20 mM HEPES, 500 mM NaCl, 5 mM MgCl_2_, 1 mM TCEP, pH 7.5 or 20 mM HEPES, 500 mM KCl, 5 mM MgCl_2_, 1 mM TCEP, pH 7.5.

### In vitro NLRP3 phase-separation assay

High concentrations of Flag-mNeonGreen-hNLRP3 protein were maintained in a high-salt buffer containing 20 mM HEPES, 500 mM NaCl/KCl, 5 mM MgCl_2_, 1 mM TCEP, pH 7.5. The hNLRP3 protein was diluted to lower salt concentration buffer to induce hNLRP3 phase separation, and MCC950 (0.1–1 mM) was added to inhibit the hNLRP3 phase separation. Alternatively, hNLRP3 protein is mixed with 1.75% PEG 8000 to induce hNLRP3 phase separation in a highly concentrated salt buffer. 1 μM Flag-mNG-hNLRP3 protein were diluted to buffer containing 20 mM HEPES, 140 mM KCl, 5 mM MgCl_2_, 1 mM TCEP, pH 7.5, and indicated compounds including amphiphilic diols (7.5%, 10%), R848 (100 μg/mL), imiquimod (100 μg/mL), cardiolipin (10–100 μM), palmitate (0.1–0.8 mM), doxorubicin (50 μM), and paclitaxel (50 μM) to induce hNLRP3 phase separation after 1 min.

### Click chemistry of 17-ODYA metabolic labeled cells

Click chemistry was performed as previously described with few modifications.^60,70^ Briefly, HEK293T cells transiently expressing Flag-NLRP3 or Flag-GFP-C^261^ or Flag-GFP-S^261^ for 24 h, or HeLa cells stably expressed with Flag-NLRP3, were labeled overnight with 50 μM palmitic acid or 50 μM 17-ODYA in DMEM supplemented with 10% FBS. The cells were washed with cold PBS (2.7 mM KCl, 137 mM NaCl, 2 mM KH_2_PO_4_, and 10 mM Na_2_HPO_4_, pH 7.4) and resuspended in lysis buffer (Tris-HCl, pH 7.5, 150 mM NaCl, 1 mM MgCl_2_, 1% NP-40, 4% SDS, and 10% glycerol) with protease inhibitor (B14001, Bimake). Samples were solubilized at RT for 30 min with gentle mixing, then centrifuged at 25,000× *g* for 60–90 min at 4 °C to reduce the concentrations of SDS and genomic DNA. Supernatants were incubated with 50 mM NEM at RT for 3 h. Subsequently, supernatants were incubated with Flag beads overnight at 4 °C with gentle mixing. After incubation, beads were washed three times with PBS; Cu^+^-assisted click reactions were performed as previously described. Briefly, beads were incubated in 50 μL of PBS supplemented with 200 μM biotin-PEG3-azide (stock solution: 20 mM TBTA in dimethyl sulfoxide (DMSO)), 100 μM TBTA (stock solution: 10 mM TBTA in 80% tert-butanol/20% DMSO), 1 mM CuSO_4_ (stock solution: 100 mM CuSO_4_ in PBS, prepared immediately before use), and 1 mM TCEP (stock solution: 50 mM TCEP in H_2_O adjusted to pH 7.0 with NaOH) at RT for 1 h with gentle mixing. Then, the beads were washed five times with cold PBS, resuspended in SDS loading buffer with 100 mM TCEP, and heated at 95 °C for 10 min. Half of the mixture was also treated with 500 μM hydroxylamine (stock solution: 5 M hydroxylamine, pH 7.4) and heated for another 5 min at 95 °C to remove S-palmitoylation. Samples were then analyzed by immunoblotting using Streptavidin Protein, DyLight™ 800 and anti-Flag antibody.

### ABE and APE assays

ABE^60,71^ and APE^72^ assays were performed as previously described with few modifications. Cells were washed with cold PBS and resuspended in lysis buffer (Tris-HCl, pH 7.5, 150 mM NaCl, 1 mM MgCl_2_, 1% NP-40, 4% SDS, and 10% glycerol) with protease inhibitor (B14001, Bimake) and 15 mM TCEP. Samples were solubilized at RT for 2 h with gentle mixing, then centrifuged at 25,000× *g* for 60–90 min at 4 °C to reduce the concentrations of genomic DNA and SDS. Supernatants were incubated with 50 mM NEM at RT for 3 h. Subsequently, the supernatants were incubated with Flag beads overnight at 4 °C with gentle mixing. Then, the beads were washed five times with reaction buffer (50 mM Tris-HCl 150 mM NaCl, 1 mM MgCl_2_, 1% NP-40, and 10% glycerol) at pH 7.5 and incubated with 0.2% MMTS at RT for 3 h. Next, the beads were washed five times with reaction buffer at pH 7.5 and four times with reaction buffer at pH 7.2. Each sample was equally divided in half; one part of beads were incubated with freshly prepared hydroxylamine-containing reaction buffer at pH 7.2 at RT (+HAM), the other part of beads omitting the hydroxylamine cleavage step (–HAM), then all beads were washed four times with reaction buffer at pH 7.2 and three times with reaction buffer at pH 6.2. For the ABE assay, beads were incubated with 3 μM biotin-BMCC-containing reaction buffer at pH 6.2 for 1 h at 4 °C. For the APE assay, beads were incubated with 1 mM mPEG-MAL (10 kD, S01-M-10K, Solarbio)-containing reaction buffer at pH 6.2 at RT for 2 h. Samples were then analyzed by immunoblotting using Streptavidin Protein, DyLight™ 800 and anti-Flag antibody.

### Detection of endogenous NLRP3 palmitoylation by ABE assay

The ABE assay^59,73^ was performed as previously described with few modifications. THP-1 cells were washed with cold PBS and resuspended in 500 μL of buffer (50 mM Tris-HCl 150 mM NaCl, 1 mM MgCl_2_, 1% NP-40, and 10% glycerol, pH 7.5) with protease inhibitor (B14001, Bimake) for 60 min at 4 °C, and then centrifuged at 12,000× *g* for 10 min at 4 °C. Supernatants were mixed with 500 μL of buffer (100 mM Tris-HCl, pH 7.2, 5 mM EDTA, 150 mM NaCl, and 5% SDS) and 15 mM TCEP, and samples were solubilized at RT for 1 h with gentle mixing. The samples were incubated with 0.2% MMTS at 40 °C for 3 h. Proteins were precipitated from samples using water/chloroform/methanol (v/v 3:1:4), and then washed twice with methanol and briefly air-dried. Next, the proteins were suspended in 1 mL of buffer (100 mM Tris-HCl, pH 7.2, 5 mM EDTA, 150 mM NaCl, and 2.5% SDS) with 50 mM N-ethylmaleimide (NEM) at RT for 3 h. Samples were subjected to protein precipitation and solubilization two more times to remove NEM. The samples were dissolved in 1 mL of buffer (100 mM Tris-HCl, pH 7.2, 5 mM EDTA, 150 mM NaCl, and 2.5% SDS) with 2 mM biotin-HPDP by gentle mixing at RT. Then, the samples were equally divided into two tubes and incubated with 0.5 mL of 1 M NaCl (negative control) or 1 M hydroxylamine (pH 7.2) at RT for 3 h, respectively. The samples were subjected to protein precipitation and solubilization twice to remove biotin-HPDP. The proteins were resuspended and dissolved in 200 μL of buffer (100 mM Tris-HCl, pH 7.2, 2% SDS, 8 M urea, and 5 mM EDTA). Twenty-microliter aliquots were used as input controls; the remaining 180 μL were diluted with PBS (1:10), then incubated with 20 μL of streptavidin beads for 12 h at 4 °C with gentle mixing. The beads were washed three times with 0.1% SDS in PBS, then heated at 95 °C for 10 min. Finally, they were analyzed by immunoblotting using anti-NLRP3 antibody.

### In vivo mouse model of lethal LPS-induced endotoxic shock

The lethal LPS-induced endotoxic shock model^26,74–77^ was performed as previously described with few modifications. Eight-week-old WT or *Zdhhc7*^−/−^ mice were randomly divided into two groups; they were subjected to intraperitoneal injection of PBS or LPS (22.5 mg/kg in PBS) to determine the effects of ZDHHC7 deletion. Eight-week-old mice were randomly divided into three groups to determine the effects of 2-BP (palmitoylation inhibitor): (i) WT + vehicle + PBS, (ii) WT + vehicle + LPS (22.5 mg/kg in PBS), and (iii) WT + 2-BP + LPS. Vehicle (5% DMSO + 5% Kolliphor^®^ EL + 90% saline) or 2-BP (50 mg/kg in 5% DMSO + 5% Kolliphor^®^ EL + 90% saline) was intraperitoneally injected before LPS challenge for 24 h and 1 h. Blood samples were collected at 12 h after LPS injection, then centrifuged at 1000× *g* for 20 min; serum aliquots were stored at –80 °C. For survival data collection, vehicle (5% DMSO + 5% Kolliphor® EL + 90% saline) or 2-BP (50 mg/kg in 5% DMSO + 5% Kolliphor® EL + 90% saline) was intraperitoneally injected before LPS challenge for 24 h and 1 h, and after LPS challenge for 24 h. Mice were monitored at 6 h intervals for 6 days.

### CETSA assay

The CETSA assay^78^ was performed as previously described with few modifications. HEK293T cells were transfected with plasmid expressing Flag-hNLRP3. After 24 h, cells were collected by centrifugation, washed with PBS, and resuspended in 0.5 mL CETSA buffer (10 mM Tris-HCl, 250 mM NaCl, PH 7.4) with protease inhibitor, and then frozen in liquid nitrogen. After two thawing-frozen cycles, the samples were centrifuged, and the supernatant was transferred. The supernatant was mixed with R848 (100 μg/mL), imiquimod (100 μg/mL), CL097 (100 μg/mL), palmitate (200 μM, 500 μM), or cardiolipin (20 μM, 50 μM) for 15 min. Then the tubes were subjected to a specified temperature for 3 min, incubated at RT for 3 min and then centrifuged at 25,000× *g* for 30 min at 4 °C. The supernatant was transferred and mixed with SDS loading buffer, heated at 95 °C for 6 min, and analyzed by immunoblotting.

### NLRP3 solubility measurement

The solubility of proteins was measured based on reported procedures^79^ with few modifications. THP-1 or expressed Flag-hNLRP3 HEK293T cells were collected in ice-cold PBS and sheared through a needle to get the cell lysates. The samples were centrifuged, and the supernatant was transferred. The supernatant was mixed with indicated concentration of ammonium sulfate, 1,4-BD, 1,6-HD, 1,2-PD, or 1,5-PD for 2 h at 4 °C and then centrifuged at 25,000× *g* for 30 min at 4 °C to get the precipitate. The pellet was mixed with SDS loading buffer, heated at 95 °C for 10 min, and analyzed by immunoblotting.

### Quantitation of cytokine production by ELISA

Mouse IL-1β (EK201B/3-96, Multi Sciences), mouse TNF-α (88-7324-22, Invitrogen), human IL-1β (88-7261-22, Invitrogen), and mouse IL-6 (88-7064-22, Invitrogen) in serum were measured as indicated by the manufacturer’s protocols.

### Statistical analysis

Data are presented as means ± SD. The number of individual experiments or mice is indicated in the figures or figure legends. The two-tailed Student’s *t*-test was employed to make comparisons between two groups. For analyzing the survival of mice, the Mantel–Cox test was used. All analyses were carried out using GraphPad Prism 9. In all figures, significance was defined as follows: *P < 0.05, **P < 0.01, ***P < 0.001. *P* values exceeding 0.05 were regarded as not significant.

## Supplementary information

Supplementary information, Fig. S1

Supplementary information, Fig. S2

Supplementary information, Fig. S3

Supplementary information, Fig. S4

Supplementary information, Fig. S5

Supplementary information, Fig. S6

Supplementary information, Fig. S7

Supplementary information, Fig. S8

Supplementary information, Fig. S9

Supplementary information, Fig. S10

Supplementary information, Table S1

Supplementary information, Video S1

Supplementary information, Video S2

Supplementary information, Video S3

Supplementary information, Video S4

Supplementary information, Video S5

## Data Availability

All data are available in the manuscript or supplementary materials.
