# Supplementary material for: Signal-induced NLRP3 phase separation initiates inflammasome activation
Source: Cell Res. 2025 Apr 1;35(6):437–52. doi: 10.1038/s41422-025-01096-6 (PMC12134225; doi:10.1038/s41422-025-01096-6)
Supplement: Supplementary file 1 — Supplementary information, Fig. S1 [file 41422_2025_1096_MOESM1_ESM.pdf]

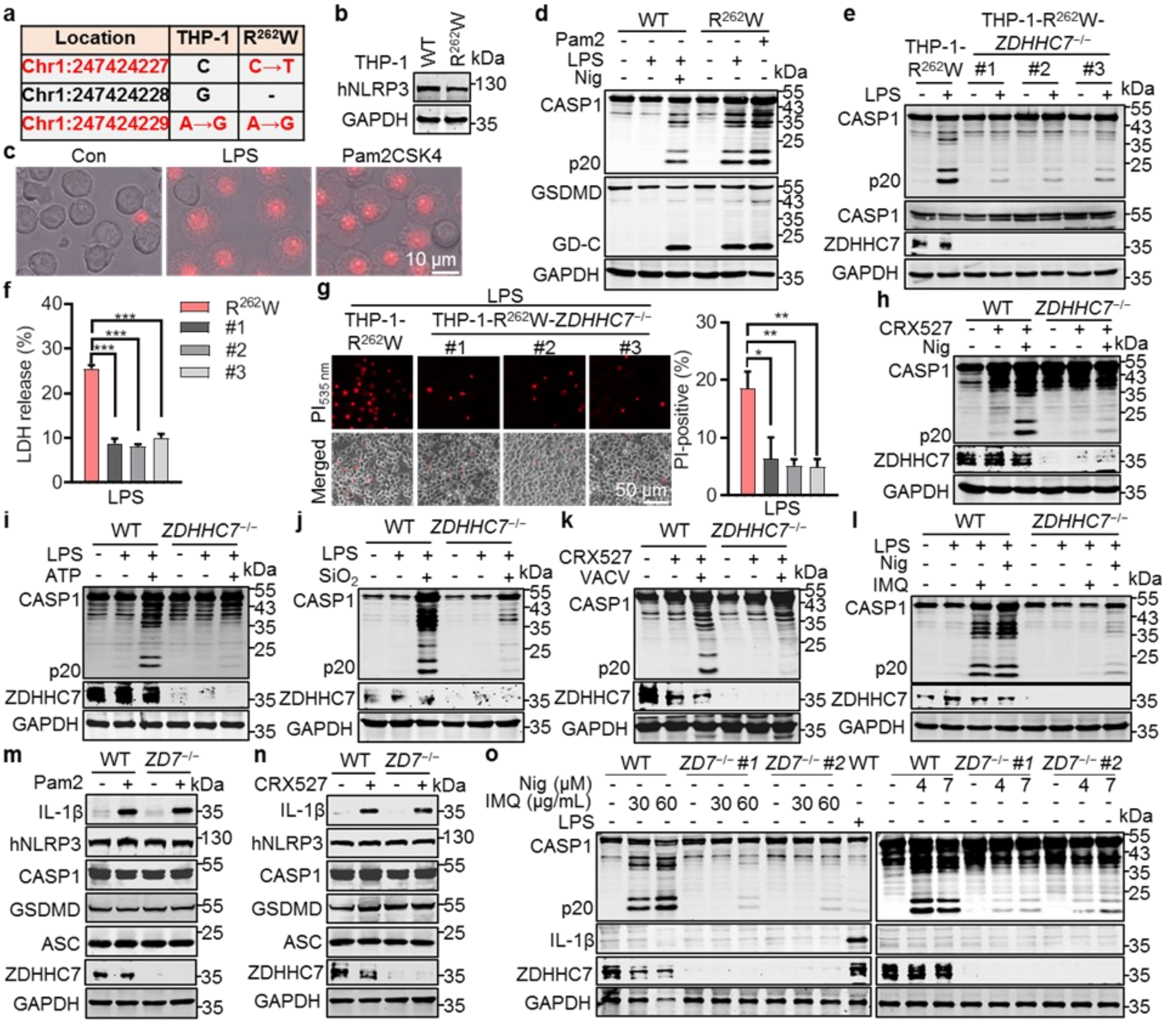

Supplementary information, Fig. S1 ZDHHC7 is required for NLRP3 activation. a, **b**, The sequence information (a) and NLRP3 expression level (b) of the WT and R^{262}W THP-1 cells. **c**, Pyroptosis in THP-1 R^{262}W cells by PI staining. In order to analyze the effects of different priming signals, cells were treated with 1 μg/mL LPS or 10 ng/mL Pam2CSK4 for 1 h before PI staining. **d**, NLRP3 activation in WT and R^{262}W THP-1 cells. Cells were pretreated with 1 μg/mL LPS or 10 ng/mL Pam2CSK4 for 3 h, followed by 4 μM nigericin for 1 h or no treatment. **e**, **f**, NLRP3 activation (e) and LDH release (f) in R^{262}W THP-1 and R^{262}W-*ZDHHC7*$^{-/-}$ cells after being treated with 1 μg/mL LPS for 1 h. **g**, Pyroptosis (left) and PI positive cells (right) in R^{262}W THP-1 and R^{262}W-*ZDHHC7*$^{-/-}$ cells as treated in (e). **h-l**, NLRP3 activation in the WT and *ZDHHC7*$^{-/-}$ THP-1 cells, priming with CRX527 (h, k) or LPS (i, j, and l). NLRP3 activation in WT and *ZDHHC7*$^{-/-}$ THP-1 cells, activated by nigericin (h), ATP (i), SiO_2 (j), VACV (k) or imiquimod (l). Cells were primed with LPS (1 μg/mL) or CRX527 (2 μg/mL) for 3 h, followed by nigericin (4 μM) for 1 h, ATP (5 mM) for 1 h, SiO_2 (500 μg/mL) for 4 h, VACV (M.O.I. = 0.1) infection for 4 h, or imiquimod (30 μg/mL) for 1.5 h. Different priming and activation stimuli were used to analyze the effect of ZDHHC7 on NLRP3 inflammasome activation. **m**, **n**, Expression level of indicated NLRP3 inflammasome components in the indicated cells treated with Pam2CSK4 (m) or CRX527 (n). *ZD7*$^{-/-}$,

ZDHHC7$^{-/-}$. **o**, Priming-independent NLRP3 activation in the indicated cells. Cells were treated with nigericin (4 μM or 7 μM) or imiquimod (30 μg/mL or 60 μg/mL) for 1 h. Statistical significance was indicated as follows: *$P < 0.05$, **$P < 0.01$, ***$P < 0.001$.
