# Supplementary material for: Signal-induced NLRP3 phase separation initiates inflammasome activation
Source: Cell Res. 2025 Apr 1;35(6):437–52. doi: 10.1038/s41422-025-01096-6 (PMC12134225; doi:10.1038/s41422-025-01096-6)
Supplement: Supplementary file 2 — Supplementary information, Fig. S2 [file 41422_2025_1096_MOESM2_ESM.pdf]

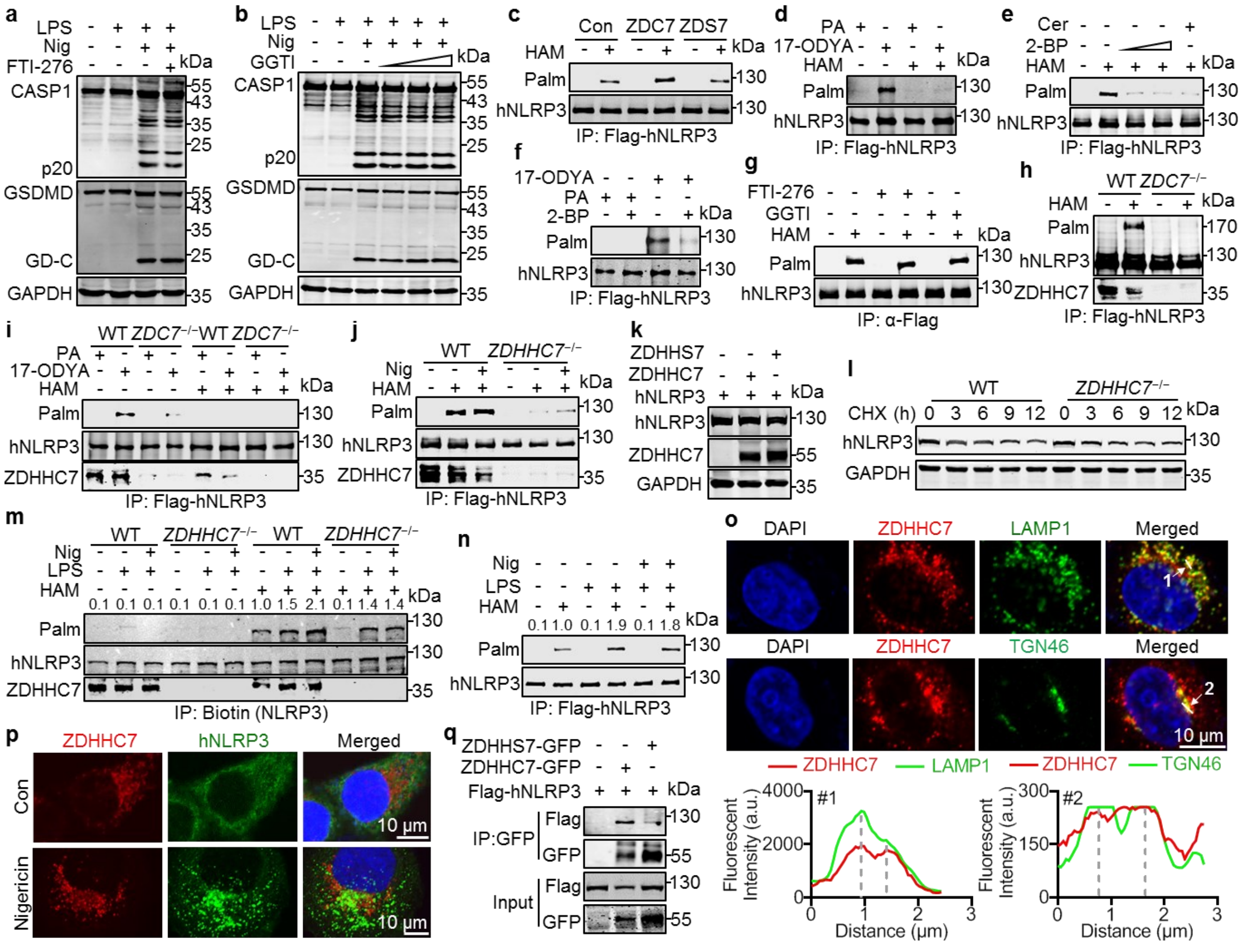

Supplementary information, Fig. S2 NLRP3 is palmitoylated by ZDHHC7. a, **b**, NLRP3 activation in THP-1 cells in the presence of 20 nM FTI-276 (a) or GGTI-2133 (5, 10, 20 μM) (b). Cells were pretreated with 1 μg/mL LPS for 2.5 h, then treated with indicated inhibitors for 30 min, followed by 4 μM nigericin treatment for another 1 h. **c**, Palmitoylation of Flag-hNLRP3 in HEK293T cells with ZDHHC7 (ZDC7) or ZDHHS7 (ZDS7) expression was detected by ABE assay. **d**, Palmitoylation of Flag-hNLRP3 in HEK293T cells was detected by click chemistry. **e-g**, HEK293T cells expressing Flag-hNLRP3 were treated overnight with 2-BP (50 μM, 100 μM; e, f), Cerulenin (Cer, 10 μg/mL; e), FTI-276 (20 nM; g) and GGTI-2133 (10 μM; g) as indicated. NLRP3 palmitoylation level was detected by ABE assay (e, g) or click chemistry (f). **h**, **i**, Palmitoylation of hNLRP3 in the WT, *ZDHHC7*$^{-/-}$, or *ZDHHC7*$^{-/-}$ HeLa cells reconstituted with ZDHHC7 or ZDHHS7 was detected by APE assay (h) or click chemistry (i). **j**, Palmitoylation of Flag-hNLRP3 in WT or *ZDHHC7*$^{-/-}$ HeLa cells was detected by ABE assay. Cells were treated by 8 μM nigericin for 1 h or not. **k**, **l**, Immunoblotting analysis of hNLRP3 protein in HEK293T with ZDHHC7 or ZDHHS7 expression (k) or WT and *ZDHHC7*$^{-/-}$ THP-1 cells treated with cycloheximide (CHX, 25 μg/mL, l) with indicated times. **m**, **n**, Palmitoylation of endogenous hNLRP3 in the WT or *ZDHHC7*$^{-/-}$ THP-1 cells (m) or Flag-hNLRP3 in *PYCARD*$^{-/-}$ THP-1 cells (n) was detected by

ABE assay. **o**, Images (up) and colocalization analysis (bottom) of HeLa cells stably expressing ZDHHC7-mCherry, immunostained with an anti-LAMP1 or anti-TGN46 antibody. White arrows indicated colocalized hNLRP3 and LAMP1 or TGN46. Quantitative analysis of co-localization along a white line was shown. **p**, Images of HeLa cells stably expressing ZDHHC7-mCherry and mNG-hNLRP3, immunostained with DAPI. **q**, The interaction between hNLRP3 and ZDHHC7 or ZDHHS7 expressed in HEK293T cells.
