# Supplementary material for: Signal-induced NLRP3 phase separation initiates inflammasome activation
Source: Cell Res. 2025 Apr 1;35(6):437–52. doi: 10.1038/s41422-025-01096-6 (PMC12134225; doi:10.1038/s41422-025-01096-6)
Supplement: Supplementary file 3 — Supplementary information, Fig. S3 [file 41422_2025_1096_MOESM3_ESM.pdf]

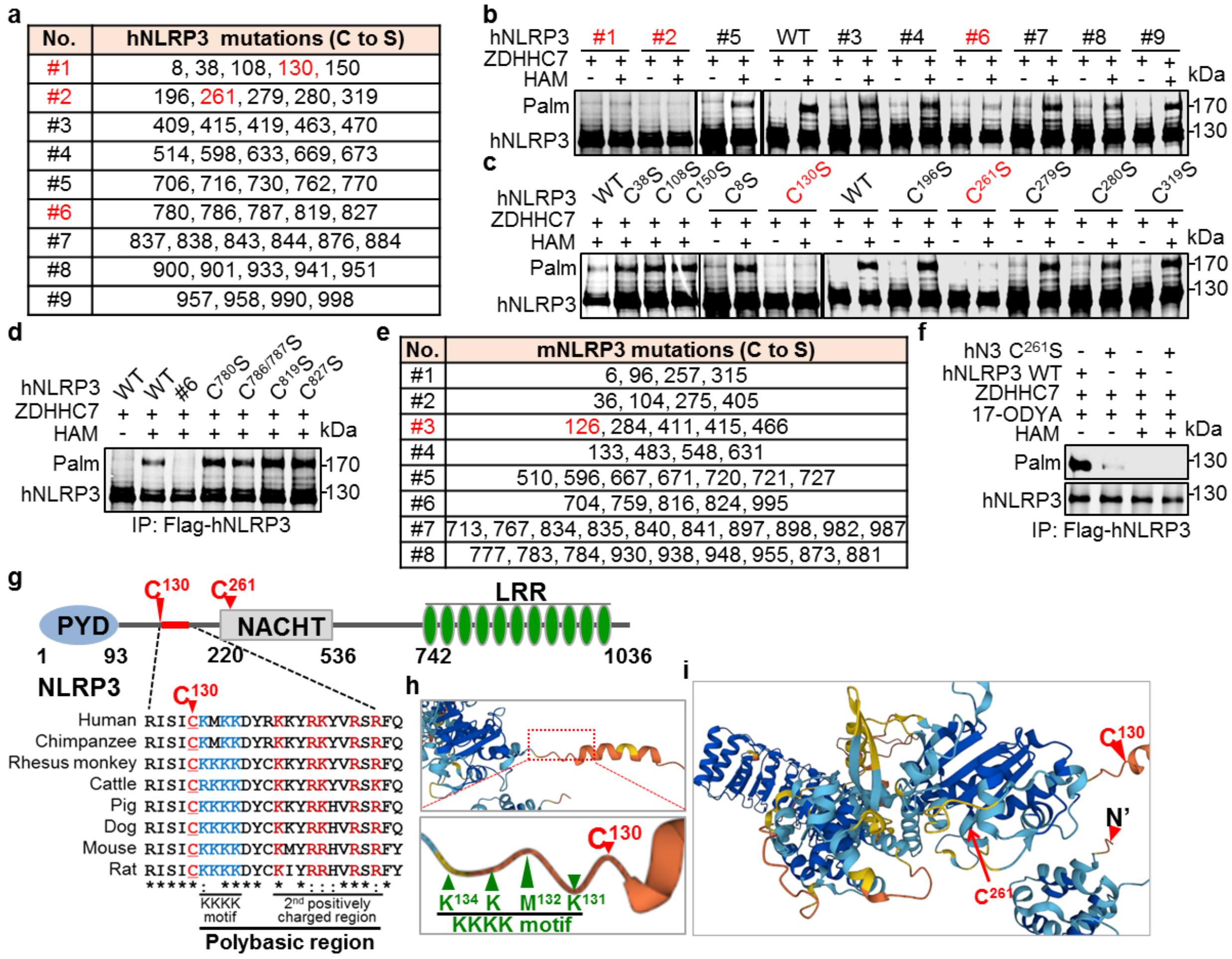

No.	hNLRP3 mutations (C to S)
#1	8, 38, 108, 130, 150
#2	196, 261, 279, 280, 319
#3	409, 415, 419, 463, 470
#4	514, 598, 633, 669, 673
#5	706, 716, 730, 762, 770
#6	780, 786, 787, 819, 827
#7	837, 838, 843, 844, 876, 884
#8	900, 901, 933, 941, 951
#9	957, 958, 990, 998

No.	mNLRP3 mutations (C to S)
#1	6, 96, 257, 315
#2	36, 104, 275, 405
#3	126, 284, 411, 415, 466
#4	133, 483, 548, 631
#5	510, 596, 667, 671, 720, 721, 727
#6	704, 759, 816, 824, 995
#7	713, 767, 834, 835, 840, 841, 897, 898, 982, 987
#8	777, 783, 784, 930, 938, 948, 955, 873, 881

Supplementary information, Fig. S3 Mapping ZDHHC7-dependent NLRP3 palmitoylation sites. **a**, Information of different numbered hNLRP3 mutations. **b-d**, Palmitoylation of Flag-hNLRP3 expressed in HEK293T cells with indicated mutations was detected by APE assay. **e**, Information of different numbered mNLRP3 mutations. **f**, Palmitoylation of Flag-hNLRP3 WT and Flag-hNLRP3 $C^{261}S$ expressed in HEK293T cells was detected by click chemistry. **g**, The domain and sequence information of aligned NLRP3. **h**, **i**, Localization and position of C^{130} with enlarged KKKK motif (h) and C^{261} (i) in the predicted structure of hNLRP3 provided by the AlphaFold DB.
