# Supplementary material for: Signal-induced NLRP3 phase separation initiates inflammasome activation
Source: Cell Res. 2025 Apr 1;35(6):437–52. doi: 10.1038/s41422-025-01096-6 (PMC12134225; doi:10.1038/s41422-025-01096-6)
Supplement: Supplementary file 4 — Supplementary information, Fig. S4 [file 41422_2025_1096_MOESM4_ESM.pdf]

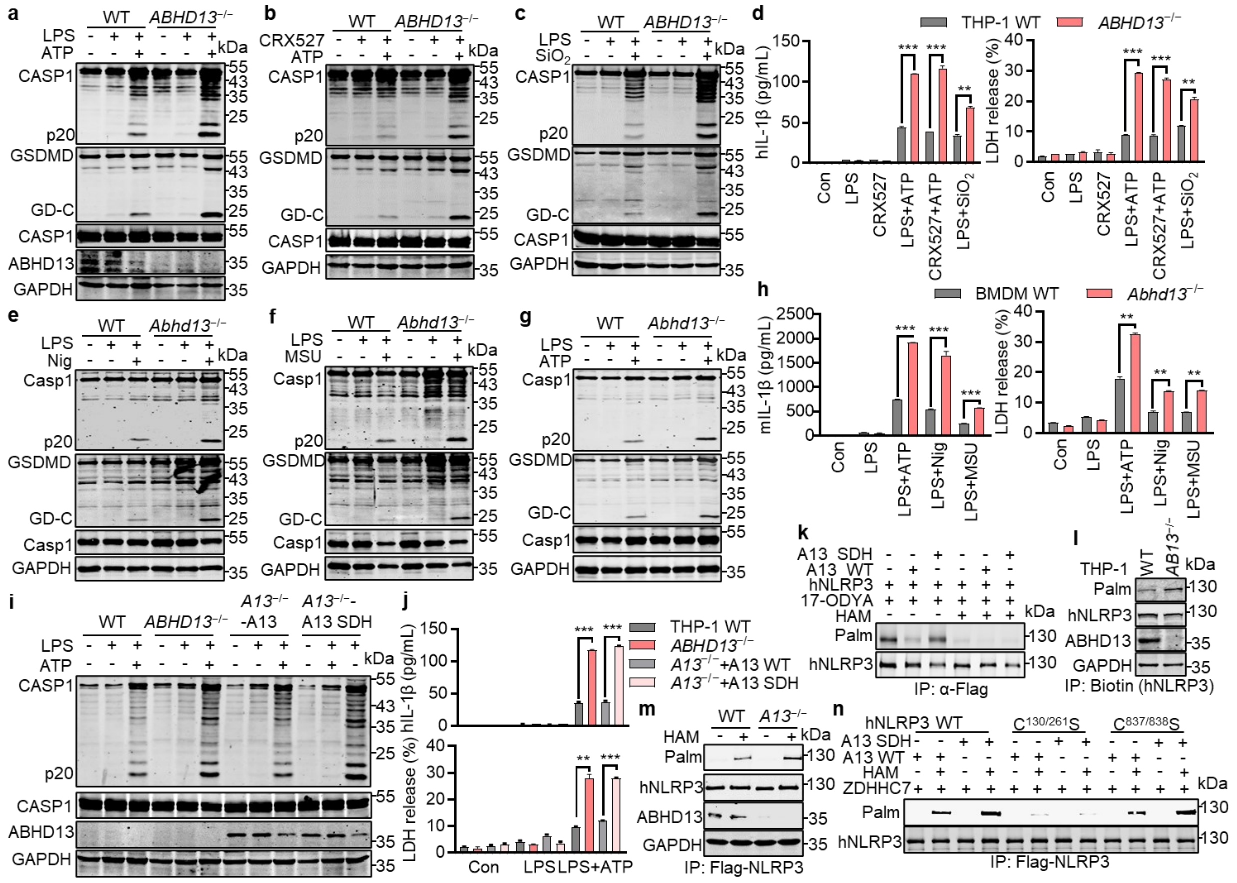

Supplementary information, Fig. S4 ABHD13 depalmitoylates and negatively regulates NLRP3. a-c, hNLRP3 activation in the WT and *ABHD13*$^{-/-}$ THP-1 cells. Cells were primed with LPS (1 µg/mL) (a, c) or CRX527 (2 µg/mL) (b) for 3 h, followed by ATP (5 mM) treatment for 1 h (a, b) or SiO_2 (250 µg/mL) treatment for 4 h (c). **d**, hIL-1β (left) and LDH release (right) in THP-1 WT or *ABHD13*$^{-/-}$ cells. Cells were treated as in (a–c). **e-g**, mNLRP3 activation in the WT or *Abhd13*$^{-/-}$ BMDMs. Cells were primed with LPS (1 µg/mL) for 3 h, followed by nigericin (4 µM) treatment for 1 h (e), MSU (250 µg/mL) treatment for 4 h (f), or ATP (5 mM) treatment for 1 h (g). **h**, mIL-1β (left) and LDH release (right) in BMDM WT or *Abhd13*$^{-/-}$ cells. Cells were treated as in (e–g). **i**, **j**, hNLRP3 activation (i) or hIL-1β and LDH release (j) in the WT, *ABHD13*$^{-/-}$, and *ABHD13*$^{-/-}$ THP-1 cells reconstituted with ABHD13 WT (*A13*$^{-/-}$-A13) or ABHD13 SDH (*A13*$^{-/-}$-SDH, S^{193}A/D^{268}A/H^{298}A). Cells were treated as in (a). **k**, Palmitoylation of hNLRP3 in HEK293T cells co-expressed with WT ABHD13 or SDH mutation was detected by click chemistry. **l**, Palmitoylation of endogenous hNLRP3 in the WT or *ABHD13*$^{-/-}$ THP-1 cells was detected by ABE assay. **m**, Palmitoylation of hNLRP3 in the WT or *ABHD13*$^{-/-}$ HeLa cells stably expressing Flag-hNLRP3 was detected by ABE assay. **n**, Palmitoylation of indicated hNLRP3 mutants in HEK293T cells co-expressed with WT ABHD13 or SDH mutation was detected by ABE assay. Statistical significance was indicated as follows: **$P < 0.01$, ***$P < 0.001$.
