# Supplementary material for: Signal-induced NLRP3 phase separation initiates inflammasome activation
Source: Cell Res. 2025 Apr 1;35(6):437–52. doi: 10.1038/s41422-025-01096-6 (PMC12134225; doi:10.1038/s41422-025-01096-6)
Supplement: Supplementary file 5 — Supplementary information, Fig. S5 [file 41422_2025_1096_MOESM5_ESM.pdf]

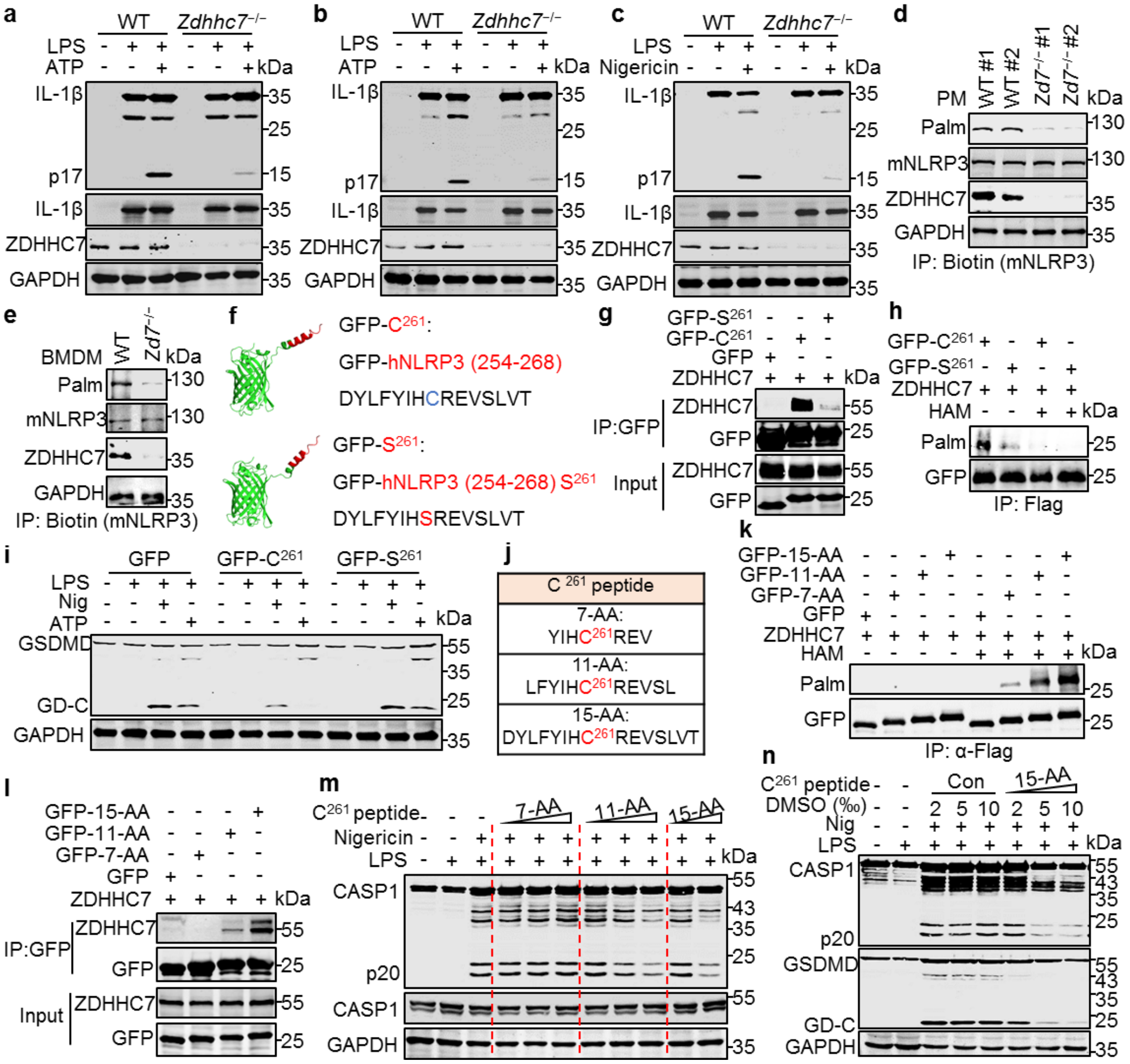

Supplementary information, Fig. S5 Targeting NLRP3 palmitoylation with a peptidic inhibitor. a-c, mNLRP3 activation in WT and *Zdhhc7*$^{-/-}$ peritoneal macrophages (a) and pulmonary fibroblasts (b, c). Cells were primed with LPS (1 μg/mL) for 3 h, followed by ATP (5 mM) treatment for 1 h (a, b) or nigericin (4 μM) treatment for 1 h (c). **d**, **e**, Palmitoylation of endogenous NLRP3 in the WT or *ABHD13*$^{-/-}$ peritoneal macrophages (PM, d) or BMDM (e) was detected by ABE assay. **f**, Structure and sequence of designed GFP-C^{261} and GFP-S^{261} peptides. **g**, The interaction between ZDHHC7-mCherry and GFP-C^{261} or GFP-S^{261} peptides. **h**, Palmitoylation of GFP-C^{261} or GFP-S^{261} peptides in HEK293T cells, co-expressed with ZDHHC7-mCherry was detected by ABE assay. **i**, NLRP3 activation in THP-1 cells stably expressing GFP, GFP-C^{261} or GFP-S^{261}. Cells were treated as in (a). **j**, Sequence of synthetic C^{261}-containing peptides. **k, l**, Palmitoylation of different peptides (k) or interaction between peptides and ZDHHC7 (l). **m, n**, NLRP3 activation in THP-1 cells after transfection with the indicated peptides dissolved in DMSO.
