# Supplementary material for: Signal-induced NLRP3 phase separation initiates inflammasome activation
Source: Cell Res. 2025 Apr 1;35(6):437–52. doi: 10.1038/s41422-025-01096-6 (PMC12134225; doi:10.1038/s41422-025-01096-6)
Supplement: Supplementary file 6 — Supplementary information, Fig. S6 [file 41422_2025_1096_MOESM6_ESM.pdf]

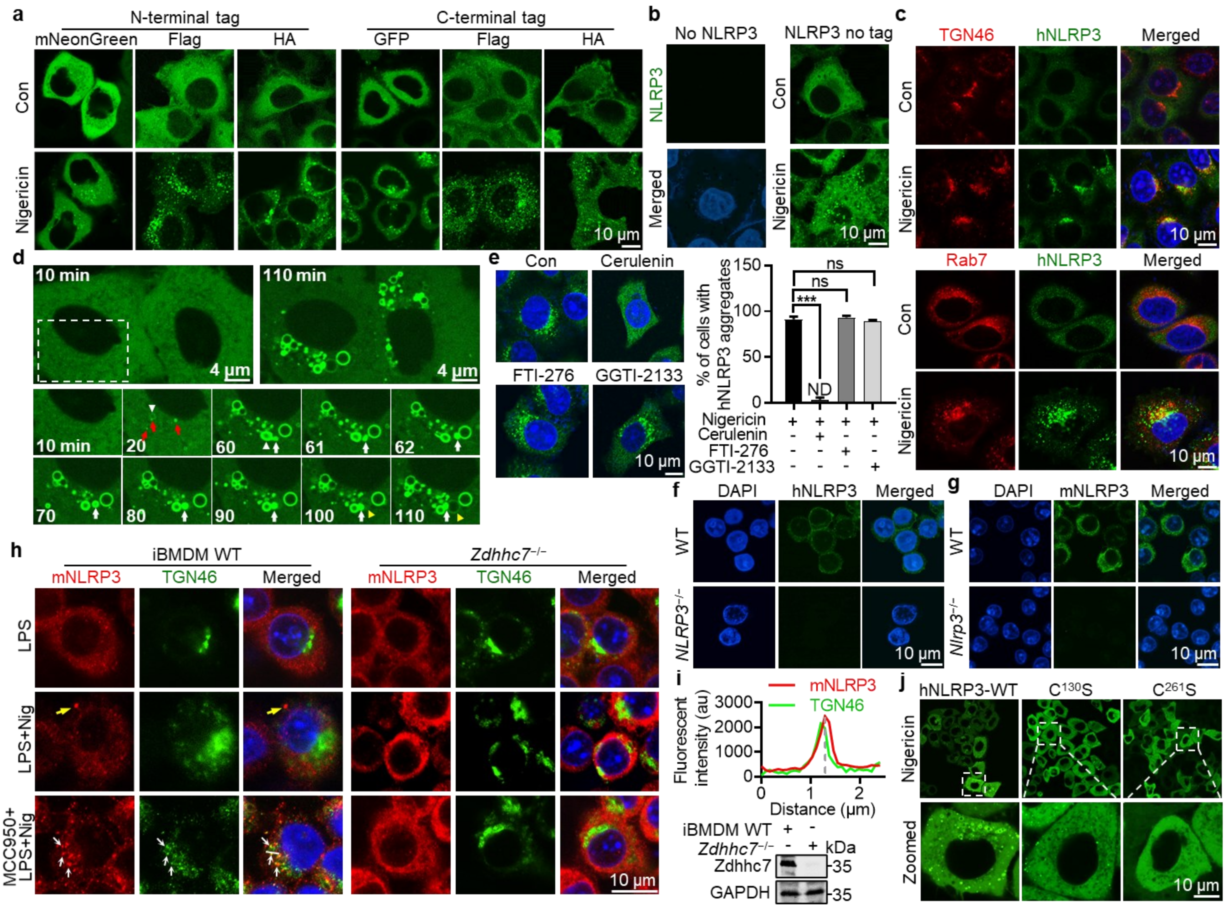

Supplementary information, Fig. S6 ZDHHC7-mediated NLRP3 palmitoylation is required for NLRP3 aggregation. **a**, Images of HeLa cells stably expressing hNLRP3 with indicated tags. For GFP or mNeonGreen tag, cells were treated with 8 μM nigericin for 1 h before live cell imaging. For Flag or HA tag, cells were treated with 8 μM nigericin for 1 h, fixed with PFA, and immunostained with indicated antibody before imaging. **b**, Images of HeLa WT cells or cells stably expressing untagged hNLRP3 after 8 μM nigericin treatment for 1 h, immunostained with an anti-NLRP3 antibody. **c**, Images of HeLa cells stably expressing Flag-hNLRP3 after 8 μM nigericin treatment for 1 h or not, immunostained with an anti-Flag antibody and anti-TGN46 (up) or anti-Rab7 (bottom) antibody. **d**, Images of HeLa cells stably expressing mNG-hNLRP3 were taken at the indicated times after 8 μM nigericin treatment. Red arrows, aggregates; white triangles, vesicles; white arrows, condensates communicate between vesicles; yellow triangles, vesicles newly formed from condensates. Scale bar, 4 μm. **e**, Images (left) and percentage of cells with aggregates (right) from HeLa cells stably expressing Flag-hNLRP3 in the presence of indicated inhibitors. Cells were pretreated with 10 μM cerulenin, 20 nM FTI-276, or 10 μM GGTI-2133 for 1 h, followed by 8 μM nigericin for another 1 h. Scale bar, 10 μm. **f**, Images of WT or *NLRP3*$^{-/-}$ THP-1 cells treated with 1 μg/mL LPS for 3 h, then fixed with PFA and immunostained with an anti-hNLRP3 antibody

(Adipogen AG-20B-0014-C100). **g**, Images of WT or *Nlrp3*$^{-/-}$ iBMDMs treated with 1 μg/mL LPS for 3 h, then fixed with PFA and immunostained with an anti-mNLRP3 antibody (Abcam ab270449). **h**, **i**, Images (h) and colocalization analysis (i) of WT and *Zdhhc7*$^{-/-}$ iBMDM cells with indicated treatment, immunostained with an anti-mNLRP3 antibody. Cells were primed with 1 μg/mL LPS for 3 h and 6 μM nigericin for 1 h in the presence of 10 μM Z-VAD-FMK to prevent pyroptosis. MCC950 (8 μM) was added 30 min before nigericin treatment. Scale bar, 10 μm. Quantitative analysis of co-localization along a white line was shown. White arrows, colocalized TGN46 and mNLRP3. Yellow arrows, NLRP3-NEK7-ASC specks. Zdhhc7 expression was analyzed by western blot using antibodies against Zdhhc7 and GAPDH (bottom). **j**, HeLa cells stably expressing mNG-hNLRP3, $C^{130}S$, or $C^{261}S$ treated with 8 μM nigericin for 1 h. Magnified pictures shown on the bottom. Statistical significance was indicated as follows: ns not significant, $^{***}P < 0.001$.
