# Supplementary material for: Signal-induced NLRP3 phase separation initiates inflammasome activation
Source: Cell Res. 2025 Apr 1;35(6):437–52. doi: 10.1038/s41422-025-01096-6 (PMC12134225; doi:10.1038/s41422-025-01096-6)
Supplement: Supplementary file 7 — Supplementary information, Fig. S7 [file 41422_2025_1096_MOESM7_ESM.pdf]

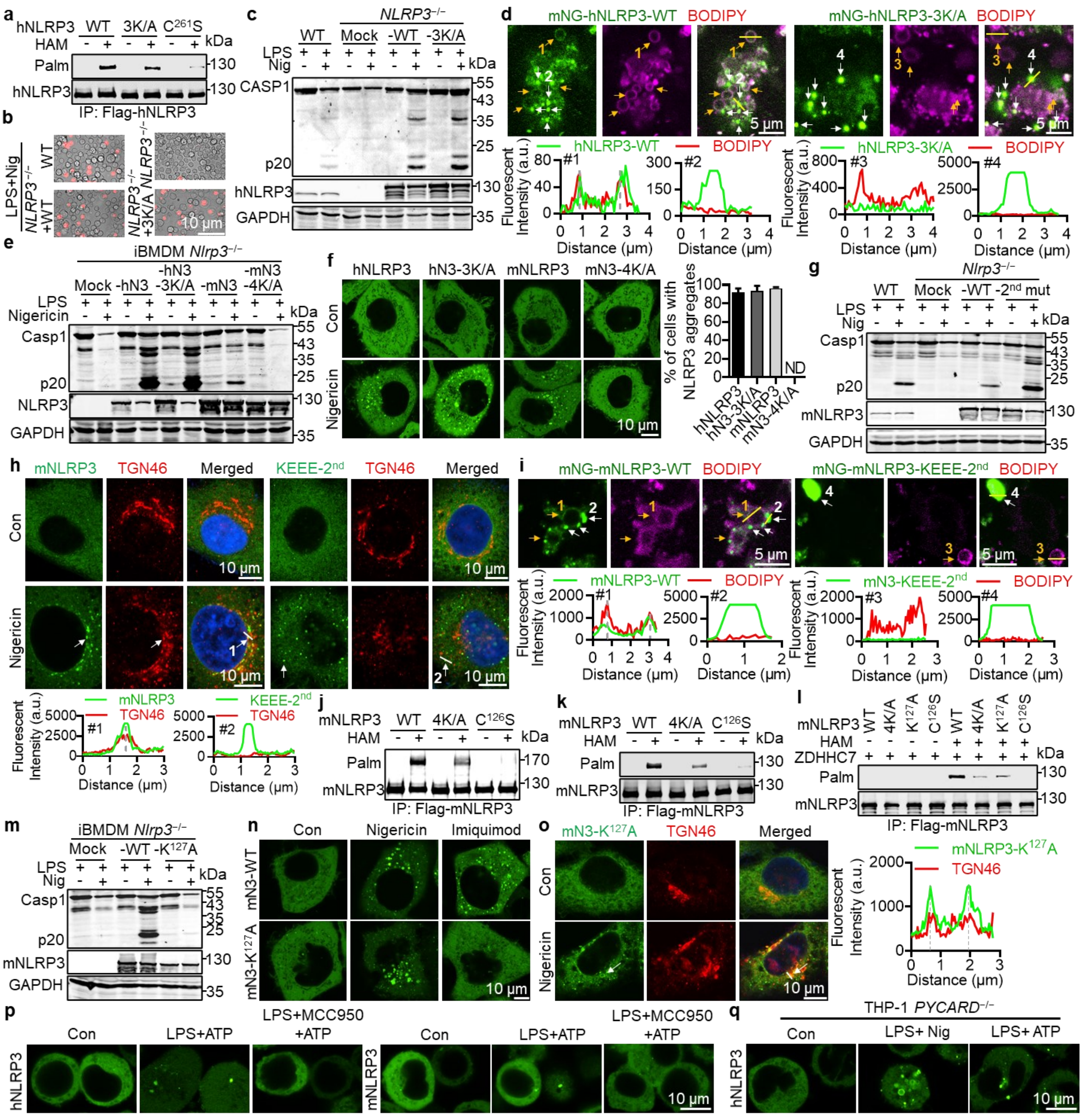

Supplementary information, Fig. S7 Condensation but not vesicle localization is required for NLRP3 activation. a, Palmitoylation of the indicated Flag-tagged hNLRP3 expressed in HEK293T cells was detected by ABE assay. **b**, **c**, Pyroptosis by PI staining (b) and NLRP3 activation (c) in WT cells and *NLRP3*$^{-/-}$ THP-1 cells reconstituted with the indicated hNLRP3-WT or hNLRP3-3K/A. Cells were treated with 1 μg/mL LPS for 3 h, followed by 4 μM nigericin treatment for another 1 h. **d**, Images (up) and colocalization analysis (bottom) of whole cell lysates from nigericin treated HeLa cells stably expressing mNG-hNLRP3-WT or mNG-hNLRP3-3K/A stained with the lipid-interacting BODIPY dye (10 μg/mL). Scale bars, 5 μm. Yellow arrows represent vesicles, white arrows represent NLRP3 condensates, and yellow lines represent the regions of colocalization analysis. **e**, NLRP3 activation in *Nlrp3*$^{-/-}$ iBMDM cells reconstituted with mNG-hNLRP3, hNLRP3-3K/A, mNLRP3, or mNLRP3-4K/A. Cells

were treated with 1 μg/mL LPS for 3 h, followed by 6 μM nigericin treatment for another 1 h. **f**, Images (left) and percentage of cells with aggregates (right) of HeLa cells stably expressing mNG-hNLRP3, hNLRP3-3K/A, mNLRP3, or mNLRP3-4K/A. Cells were treated with 8 μM nigericin for 1 h before live cell imaging. Scale bar, 10 μm. **g**, mNLRP3 activation in WT or *Nlrp3*$^{-/-}$ iBMDM cells reconstituted with indicated mutants. Cells were treated as in (e). mNLRP3-2nd mut, K^{134}M/R$^{137/138/141/143}$A. **h**, Images of HeLa cells expressing mNG-mNLRP3 WT or KEEE-2nd. Cells were treated with 8 μM nigericin for 1 h or not before being fixed and immunostained with an anti-TGN46 antibody. Quantitative analysis of co-localization along a white line was shown on the bottom. Scale bars, 10 μm. **i**, Images (up) and colocalization analysis (bottom) of whole cell lysates from nigericin treated HEK293T cells expressing mNG-mNLRP3-WT or mNG-mNLRP3-KEEE-2nd mut stained with the lipid-interacting BODIPY dye (10 μg/mL). Scale bars, 5 μm. Yellow arrows represent vesicles, white arrows represent NLRP3 condensates, and yellow lines represent the regions of colocalization analysis. **j-l,** Palmitoylation of Flag-mNLRP3 with indicated mutations expressed in B16 cells was detected by APE (j) and ABE assay (k, l). **m**, mNLRP3 activation in WT or *Nlrp3*$^{-/-}$ iBMDM cells reconstituted with indicated mutants. Cells were treated as in (e). **n**, Images of HeLa cells stably expressing mNG-mNLRP3-WT or mNG-mNLRP3-K^{127}A. Cells were treated with 8 μM nigericin or 40 μg/mL imiquimod for 1 h before live cell imaging. Scale bar, 10 μm. **o**, Images (left) and colocalization analysis (right) of HeLa cells stably expressing mNLRP3-K^{127}A with 8 μM nigericin for 1 h or not, immunostained with an anti-TGN46 antibody. Scale bar, 10 μm. White arrows indicated the colocalization between mNLRP3-K^{127}A and TGN46. Quantitative analysis of co-localization along a white line was shown. **p**, Live cell images of *Nlrp3*$^{-/-}$ iBMDMs reconstituted with mNG-hNLRP3 or mNG-mNLRP3. Cells were treated with 1 μg/mL LPS for 3 h, followed by 2.5 mM ATP for 1 h. Cells were pretreated with 10 μM MCC950 for 30 min before ATP treatment to inhibit NLRP3 phase-separation. Scale bar, 10 μm. **q**, Live cell images of *PYCARD*$^{-/-}$ THP-1 cells stably expressing mNG-hNLRP3. Cells were treated with 1 μg/mL LPS for 3 h, followed by 4 μM nigericin or 2.5 mM ATP for 1 h. Scale bar, 10 μm.
