# Supplementary material for: Signal-induced NLRP3 phase separation initiates inflammasome activation
Source: Cell Res. 2025 Apr 1;35(6):437–52. doi: 10.1038/s41422-025-01096-6 (PMC12134225; doi:10.1038/s41422-025-01096-6)
Supplement: Supplementary file 8 — Supplementary information, Fig. S8 [file 41422_2025_1096_MOESM8_ESM.pdf]

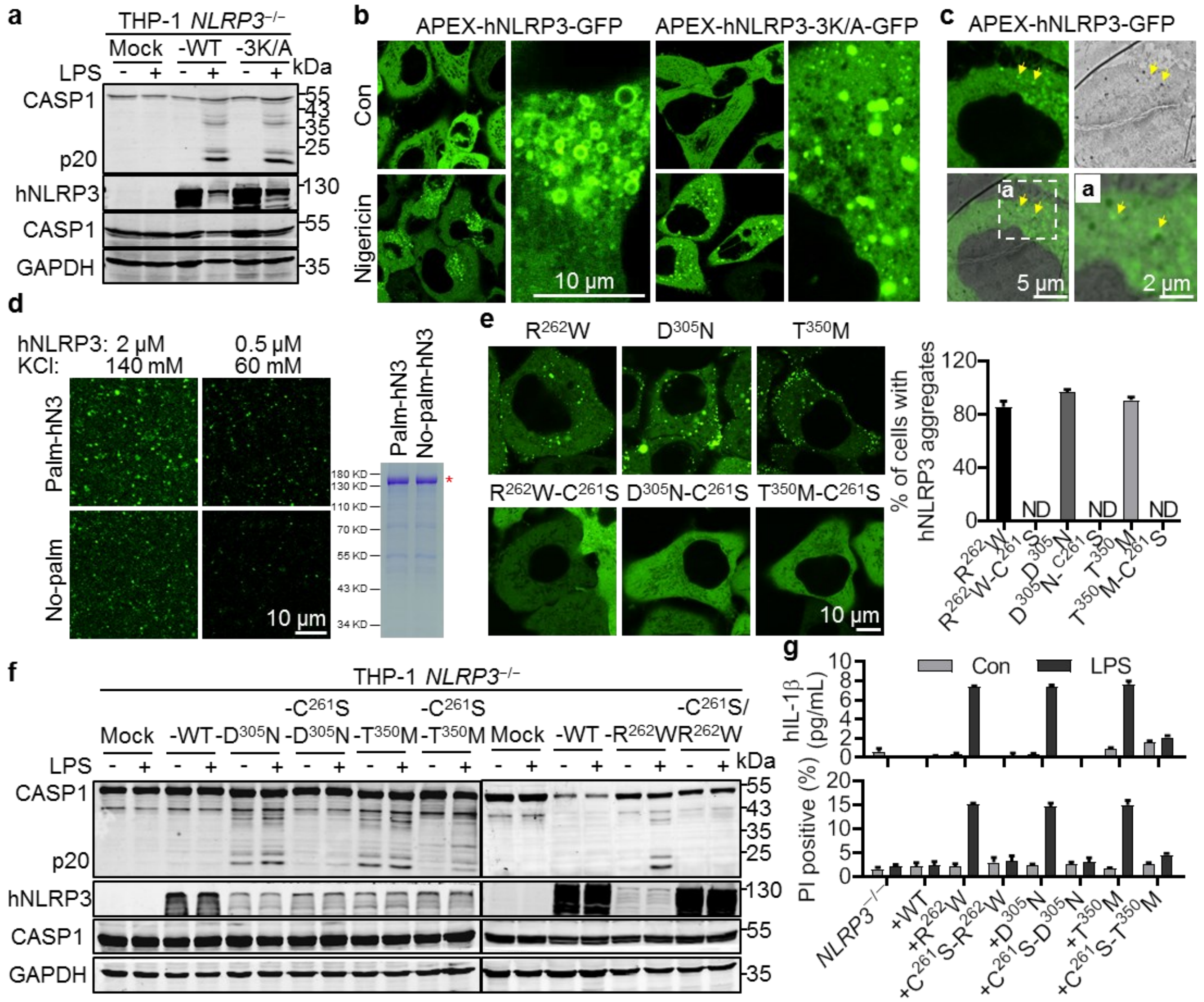

Supplementary information, Fig. S8 Palmitoylation is required for NLRP3 phase separation and activation in CAPS. **a**, NLRP3 activation in *NLRP3*$^{-/-}$ THP-1 cells reconstituted with APEX-hNLRP3-EGFP or APEX-hNLRP3-3K/A-EGFP. Cells were treated with 1 µg/mL LPS for 3 h, followed by 4 µM nigericin treatment for 1 h. **b**, Images of HeLa cells stably expressing APEX-hNLRP3-EGFP or APEX-hNLRP3-3K/A-EGFP. Magnified images are shown on the right. Cells were treated with 8 µM nigericin for 1 h or not before live cell imaging. Scale bar, 10 µm. **c**, CLEM images of HeLa cells stably expressing APEX-hNLRP3-EGFP after 8 µM nigericin treatment for 1 h. Scale bars, 5 µm or 2 µm. **d**, Condensation of purified palm-mNG-hNLRP3 or no-palm-mNG-hNLRP3 (0.5 or 2 µM) in phase-separation buffer with 60 or 140 mM KCl at room temperature for 3 min. Palm-mNG-hNLRP3 or no-palm-mNG-hNLRP3 protein were purified from the WT or *ZDHHC7*$^{-/-}$ HEK293T cells, respectively. Scale bar, 10 µm. Coomassie blue staining of purified NLRP3 protein was shown on the right. **e**, Live cell images (left) or percentage of cells with aggregates (right) in HeLa cells stably expressing indicated autoactive NLRP3 mutants and combined mutation with C^{261}S without any treatment. Scale bar, 10 µm. **f**, NLRP3 activation in *NLRP3*$^{-/-}$ THP-1 cells reconstituted with indicated mutants. Cells were treated

with 1 μg/mL LPS for 1 h or left untreated. **g**, hIL-1β release (up) or PI positive cells (bottom) in *NLRP3*$^{-/-}$ THP-1 cells reconstituted with indicated mutants. Cells were treated as in (f).
