# Supplementary material for: Signal-induced NLRP3 phase separation initiates inflammasome activation
Source: Cell Res. 2025 Apr 1;35(6):437–52. doi: 10.1038/s41422-025-01096-6 (PMC12134225; doi:10.1038/s41422-025-01096-6)
Supplement: Supplementary file 9 — Supplementary information, Fig. S9 [file 41422_2025_1096_MOESM9_ESM.pdf]

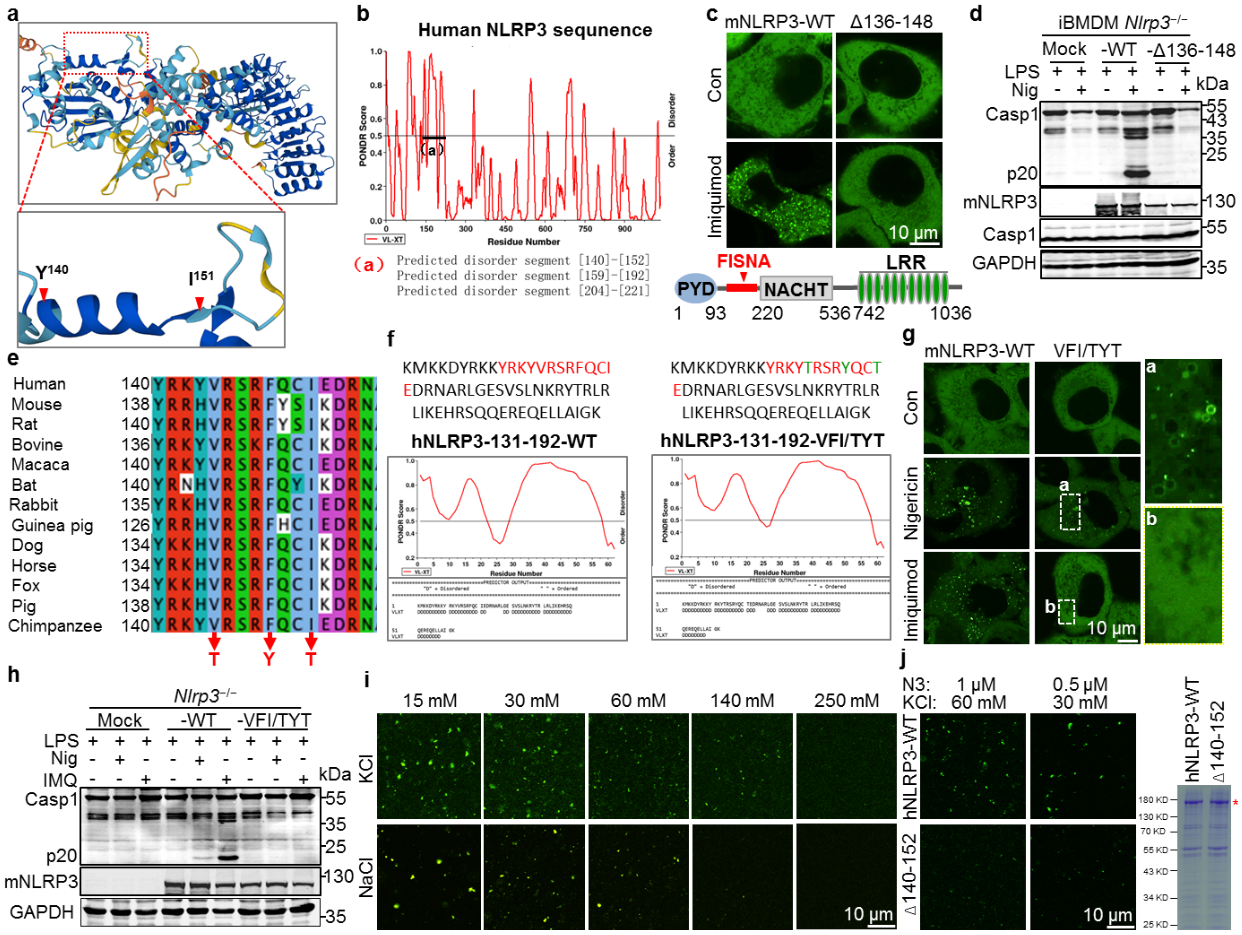

Supplementary information, Fig. S9 An IDR in the FISNA domain is required for NLRP3 phase separation. **a**, Localization and position of IDR in the predicted structure of human NLRP3 provided by the AlphaFold DB. **b**, IDR prediction of NLRP3 by PONDR VL-XT. **c**, Images of HeLa cells stably expressing mNG-mNLRP3 or Δ136-148. Cells were treated with 40 µg/mL imiquimod for 1 h or not before live cell imaging. Scale bar, 10 µm. **d**, mNLRP3 activation in *Nlrp3*$^{-/-}$ iBMDM cells reconstituted with indicated mutants. Cells were pretreated with 1 µg/mL LPS for 3 h, followed by 6 µM nigericin treatment for 1 h. **e**, The IDR region of NLRP3 is highly conserved in NLRP3 orthologues (aligned by ClustalW). **f**, IDR prediction of NLRP3 WT or VFI/TYT by PONDR VL-XT. **g**, Images of cells with mNLRP3 aggregates in HeLa cells stably expressing mNG-mNLRP3 WT or mNG-mNLRP3 VFI/TYT. Cells were treated with 8 µM nigericin or 40 µg/mL imiquimod for 1 h before live cell imaging. Scale bar, 10 µm. **h**, NLRP3 activation in iBMDM *Nlrp3*$^{-/-}$ cells reconstituted with mNG-mNLRP3 WT or VFI/TYT. Cells were treated with 1 µg/mL LPS for 3 h, followed by 6 µM nigericin or 60 µg/mL imiquimod treatment for 1 h. **i**, Condensation of purified mNG-hNLRP3 (1 µM) in phase-separation buffer with the indicated KCl or NaCl concentrations. Scale bar, 10 µm. **j**, Condensation of purified mNG-hNLRP3 WT or mNG-hNLRP3 Δ140-152 (0.5 or 1 µM) in phase-separation buffer with 30 or 60 mM KCl at room temperature for 3 min. Scale bar, 10

μm. Coomassie blue staining of purified NLRP3 protein was shown on the right.
