# Supplementary material for: Signal-induced NLRP3 phase separation initiates inflammasome activation
Source: Cell Res. 2025 Apr 1;35(6):437–52. doi: 10.1038/s41422-025-01096-6 (PMC12134225; doi:10.1038/s41422-025-01096-6)
Supplement: Supplementary file 10 — Supplementary information, Fig. S10 [file 41422_2025_1096_MOESM10_ESM.pdf]

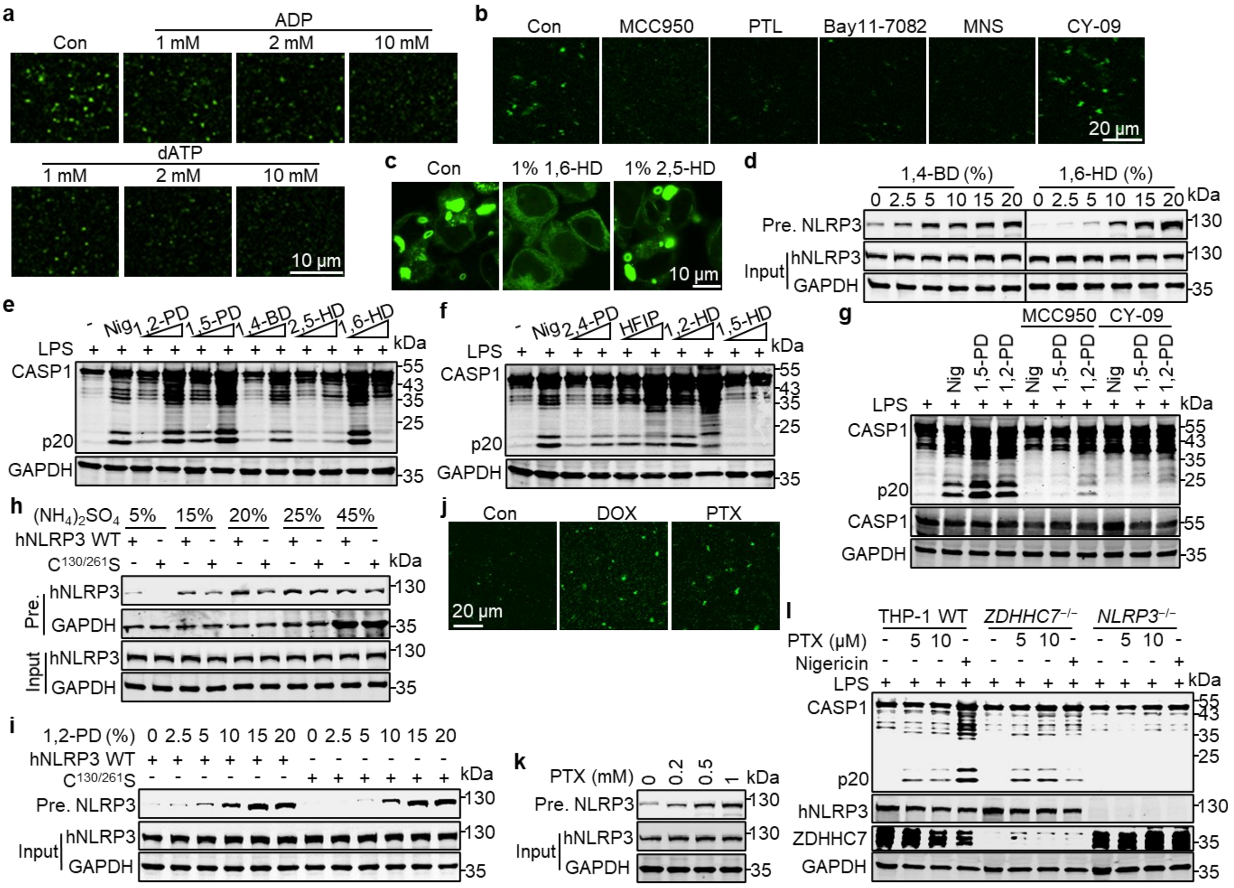

Supplementary information, Fig. S10 Amphiphilic molecules induce NLRP3 phase separation. **a**, In vitro NLRP3 (1 µM) LLPS in a phase-separation buffer with 30 mM KCl in the presence of indicated concentrations of ADP or dATP. **b**, In vitro LLPS of NLRP3 (1 µM) in a phase-separation buffer in the presence of 1% DMSO (Con), 1 mM MCC950, 0.15 mM parthenolide (PTL), 1 mM Bay11-7082, 0.1 mM MNS or 0.2 mM CY-09. **c**, Images of HEK293T cells transfection with STING-EGFP. Cells were treated with 1% 1,6-HD or 1% 2,5-HD for 1 h or not before live cell imaging. Scale bar, 10 µm. **d**, The solubility of NLRP3 in whole cell lysates of HEK293T cells expressing Flag-hNLRP3 in the presence of indicated concentration of amphiphilic di-alcohols including 1,4-BD and 1,6-HD. Precipitated NLRP3 (Pre. NLRP3) was detected by immunoblotting. **e**, **f**, NLRP3 activation in THP-1 cells treated with the indicated amphiphilic molecules. Cells were treated with 1 µg/mL LPS for 3 h, followed by 2 µM nigericin treatment for another 1 h, or cells were treated with 1 µg/mL LPS for 30 min, followed by 1,6-HD (1.2%, 2.5%), 2,5-HD (1.2%, 2.5%), 1,4-BD (1.2%, 2.5%), 1,2-PD (0.6%, 1.2%), 1,5-PD (1.2%, 2.5%), 2,4-PD (0.6%, 1.2%), 1,2-HD (0.3%, 0.6%), HFIP (0.15%, 0.3%), or 1,5-HD (0.3%, 0.6%) treatment for another 3–4 h. **g**, NLRP3 activation in THP-1 cells treated by 2 µM nigericin, 2.5% 1,5-PD or 1.2% 1,2-PD in the presence of 10 µM MCC950 or 20 µM CY-09. **h, i,** The solubility of NLRP3 in whole cell lysates of HEK293T cells expressing Flag-hNLRP3-WT or Flag-hNLRP3-C$^{130/261}$S in the presence of the indicated

concentrations of ammonium sulfate (g/ 100 ml whole cell lysate) (h) or 1,2-PD (i) was analyzed. Experiment was conducted as in (d). **j**, In vitro NLRP3 (1 μM) LLPS in a phase-separation buffer with 140 mM KCl in the presence of 100 μM doxorubicin (DOX) or 100 μM paclitaxel (PTX). Scale bar, 20 μm. **k**, NLRP3 solubility in cell lysate from HEK293T cells expressing Flag-hNLRP3 in the presence of indicated concentration of paclitaxel. Precipitated NLRP3 (Pre. NLRP3) was detected by immunoblotting. **l**, NLRP3 activation in WT, *ZDHHC7*$^{-/-}$, or *NLRP3*$^{-/-}$ THP-1 cells pretreated with 0.2 μg/mL LPS for 3 h and 2 μM nigericin for another 1 h, or pretreated with 0.2 μg/mL LPS for 30 min and paclitaxel (PTX, 5 μM, 10 μM) for another 15 h.
