# Supplementary material for: Signal-induced NLRP3 phase separation initiates inflammasome activation
Source: Cell Res. 2025 Apr 1;35(6):437–52. doi: 10.1038/s41422-025-01096-6 (PMC12134225; doi:10.1038/s41422-025-01096-6)
Supplement: Supplementary file 11 — Supplementary information, Table S1 [file 41422_2025_1096_MOESM11_ESM.pdf]

Supplementary information, Table 1 Genotyping of targeted genes in the indicated cells.

KO Cell lines	PCR Sequencing
HeLa-*ZDHHC7*$^{-/-}$	10 bp deletion (underlined) TGAAGCCCGGGGAAGTCATCTA
HeLa-*ABHD13*$^{-/-}$	11 bp deletion (underlined) CCCATGCCCACTGGCATTC; 2 bp deletion (underlined) TTATGTTCCCATGCCCACTGGCATT
THP-1-*ZDHHC7*$^{-/-}$#1	133 bp deletion (underlined) ACTCTTCATCGTCCTCCTCCTCCGAGGCTGACGTGGCTGACCGGGTCTGGTTCATCCGTGACGGCTGCGGCATGATCTGTGCTGTCATGACGTGGCTTCTGGTCGCCTATGCAGACTTCGTGGTGACTTTCGTCATGCTGC
THP-1-*ZDHHC7*$^{-/-}$#2	28 bp deletion (underlined) AAATGGATCATCACTGCCCGTGGGTGAACAATTGTG
THP-1-*ABHD13*$^{-/-}$	1 bp deletion (underlined) TTCTTTCCGATGCGTTACCTTCCT
THP-1-NLRP3-R^{262}W -*ZDHHC7*$^{-/-}$#1	59 bp deletion (underlined) TGGTCGCCTATGCAGACTTCGTGGTGACTTTCGTCATGCTGCTGCCTTCCAAAGACTTCTGGTACTC
THP-1-NLRP3-R^{262}W -*ZDHHC7*$^{-/-}$#2	25 bp deletion (underlined) ACGTGGCTTCTGGTCGCCTATGCAGACTTCGTG
THP-1-NLRP3-R^{262}W -*ZDHHC7*$^{-/-}$#3	41 bp deletion (underlined) TGTGCTGTCATGACGTGGCTTCTGGTCGCCTATGCAGACTTCGTGGTGA
iBMDM-*Zdhhc7*$^{-/-}$	1 bp deletion (underlined) GCATCCTCTTCGTCCTCCGAGACTGACATGGCAGACAGGGTGT; 2 bp deletion (underlined) TCCTCCGAGACTGACATG
iBMDM-*Nlrp3*$^{-/-}$	1 bp deletion (underlined) ACATTCCTCTATGGTATGCCAGGAGGACAGCCTTGAAGA
